# Association of SARS-CoV-2 infection with incident diabetes among U.S. Veterans in a prospective longitudinal cohort

**DOI:** 10.1371/journal.pone.0351992

**Published:** 2026-06-26

**Authors:** Liuye Huang, Tracy M. Wang, Jonathan D. Sugimoto, Kent R. Heberer, Aaron J. Baraff, Anna M. Korpak, Alexandra E. Fox, Daniel K. Morelli, Jordanna B. Midthun, Antonio Anzueto, Roger J. Bedimo, Eric Garshick, Kyong-Mi Chang, Robin L. Jump, Alaina S. Ritter, Patrick J. Danaher, McKenna C. Eastment, Stuart N. Isaacs, Elizabeth Le, Gary P. Wang, Nicholas L. Smith, Jennifer M. Ross, Javeed A. Shah, Jennifer S. Lee, Pandora L. Wander

**Affiliations:** 1 Veterans Affairs Puget Sound Health Care System, Seattle, Washington, United States of America; 2 Department of Epidemiology, School of Public Health, University of Washington, Seattle, Washington, United States of America; 3 International Vaccine Institute, Seoul, Korea; 4 Veterans Affairs Palo Alto Health Care System, Palo Alto, California, United States of America; 5 South Texas Veterans Health Care System, San Antonio, Texas, United States of America; 6 University of Texas Health Science Center at San Antonio, Texas, United States of America; 7 VA North Texas Health Care System, Dallas, Texas, United States of America; 8 University of Texas Southwestern Medical Center, Dallas, Texas, United States of America; 9 VA Boston Healthcare System, Boston, Massachusetts, United States of America; 10 Harvard Medical School, Boston, Massachusetts, United States of America; 11 Corporal Michael J. Crescenz VA Medical Center, Philadelphia, Pennsylvania, United States of America; 12 University of Pennsylvania Perelman School of Medicine, Philadelphia, Pennsylvania, United States of America; 13 Geriatric Research, Education, and Clinical Center, Pittsburgh VA Healthcare System, Pittsburgh, Pennsylvania, United States of America; 14 Division of Geriatrics, Department of Medicine, University of Pittsburgh School of Medicine, Pittsburgh, Pennsylvania, United States of America; 15 Division of Infectious Diseases, North Florida/South Georgia Veterans Health System, Gainesville, Florida, United States of America; 16 Division of Infectious Diseases and Global Medicine, Department of Medicine, University of Florida College of Medicine, Gainesville, Florida, United States of America; 17 James A. Haley Veterans’ Hospital, Tampa, Florida, United States of America; 18 Division of Infectious Disease and International Medicine, University of South Florida, Tampa, Florida, United States of America; 19 Division of Allergy and Infectious Diseases, Department of Medicine, University of Washington, Seattle, Washington, United States of America; 20 Department of Global Health, University of Washington, Seattle, Washington, United States of America; 21 School of Medicine, Stanford University, Stanford, California, United States of America; 22 Clinical Innovation and Research and Development, Veterans Affairs Palo Alto Health Care System, Palo Alto, California, United States of America; 23 Division of Endocrinology, Gerontology, and Metabolism, Department of Medicine, School of Medicine, Stanford University, Stanford, California, United States of America; 24 Division of General Internal Medicine, Department of Medicine, University of Washington, Seattle, Washington, United States of America; Gulu University, UGANDA

## Abstract

**Objective:**

To assess care-seeking patterns and incident diabetes risk following SARS-CoV-2 infection in a prospective longitudinal cohort.

**Research design and methods:**

We used data from the Veterans Health Administration (VHA)-based, prospective longitudinal study Epidemiology, Immunology, and Clinical Characteristics of COVID-19 (EPIC^3^) and electronic health records from participants with and without a history of SARS-CoV-2 infection who were free from diabetes at baseline and enrolled between June 2020 and September 2022 (n = 1,212); participants were followed prospectively for a median of approximately 4 years. We fit Cox proportional hazard models to examine associations of prior SARS-CoV-2 infection with incident diabetes. Models were adjusted for age, sex, race, smoking status, BMI, education, and comorbidities, as well as number of laboratory test days in the year prior.

**Results:**

Men comprised 79.4% of the cohort. Median age was 50.1 (SARS-CoV-2–positive) and 57.2 (SARS-CoV-2–negative) years. After accounting for time-varying SARS-CoV-2 infection status, compared to participants with a negative test, participants with a positive test had fewer days/year with clinic visits (18.2 vs. 25.4, p < 0.001), laboratory tests of any kind (3.1 vs. 4.3, p < 0.001), and glucose tests (1.8 vs. 2.8, p < 0.001) post-enrollment; however, the number of days/year on which they had HbA1c tests was not significantly different (0.7 vs. 0.7, p = 0.094); diabetes incidence was 16.1 and 21.2 per 1,000 person-years in SARS-CoV-2 positive and negative groups, respectively. SARS-CoV-2 was not associated with adjusted diabetes-free survival overall, in inpatients or in outpatients (adjusted HRs: 0.82 [95% CI, 0.51–1.35], 1.03 [0.27–3.96], and 0.79 [0.44–1.39], respectively).

**Conclusion:**

Although SARS-CoV-2–positive participants used healthcare less often than those without, HbA1c testing rates were similar. We did not replicate prior reports of higher diabetes risk after SARS-CoV-2, although small sample size may have reduced power to detect modest associations. NCT: NCT05764083.

## Introduction

Diabetes places an enormous burden on society, with a total estimated cost in the United States of more than $400 billion in 2022 [1]. SARS-CoV-2/COVID has been linked to a higher risk of incident diabetes, with pooled estimates from meta-analyses of retrospective studies suggesting a 50–80% higher risk [1–6]. Contributing mechanisms might include direct or indirect viral injury to β cells or systemic effects (e.g., insulin resistance and inflammation due to the infection itself or to treatments such as glucocorticoids) [7,8]. Because SARS-CoV-2 infection is extremely common [9], even a modest increase in diabetes risk in the context of SARS-CoV-2 infection could translate into a substantial rise in global diabetes prevalence. Accurate estimates of the relationship between SARS-CoV-2 and incident diabetes risk are needed to characterize the attributable risk of diabetes due to COVID and/or to help anticipate long-term impacts of the pandemic on glycemia and diabetes-related health care costs.

Current estimates of the risk of incident diabetes after SARS-CoV-2 are primarily derived from retrospective analyses of electronic health records (EHR) or administrative data [1–6]. Such designs may be influenced by differences in care-seeking behavior or clinical surveillance between individuals with and without recent SARS-CoV-2 infection [10]. In this context, individuals who are acutely ill might be compelled to seek care and those who can defer routine care might elect to do so. Because diabetes, particularly type 2 diabetes, is frequently asymptomatic, it is often identified not through targeted diagnostic evaluation but incidentally during routine lab testing for other indications. Thus, if individuals with and without recent COVID in retrospective datasets have different patterns in care-seeking behavior or surveillance, it raises the possibility that at least part of the observed association of SARS-CoV-2 infection with incident diabetes might be due to bias from differences in clinical access and surveillance and not true differences in diabetes incidence.

We utilized data from the Veterans Health Administration (VHA)-based prospective longitudinal observational study Epidemiology, Immunology and Clinical Characteristics of COVID-19 (EPIC^3^) [11] to examine patterns of clinical care-seeking behavior and to estimate the relationship between SARS-CoV-2 infection and incident diabetes. We tested the hypothesis that clinical care-seeking behaviors differ between participants with and without recent SARS-CoV-2 at the time of their enrollment. We further hypothesized that the risk of incident diabetes would differ between individuals with and without recent SARS-CoV-2.

## Materials and methods

### Study population

**Fig 1:**
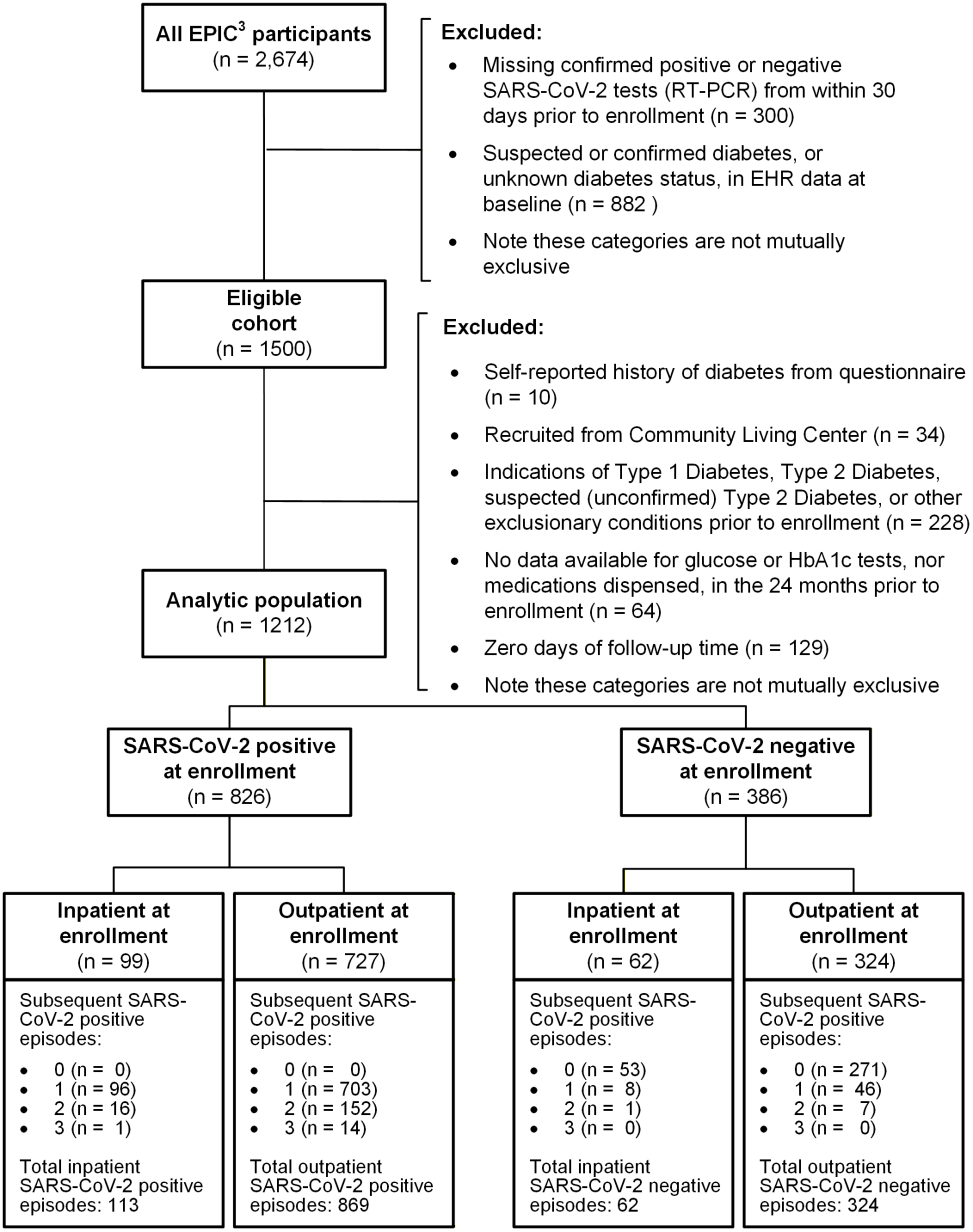
Flow diagram of participants included in the analytic population.

This current analysis utilized data from EPIC^3^ and the VHA Corporate Data Warehouse (CDW). EPIC^3^ is a prospective longitudinal observational cohort study that enrolled U.S. Veterans who were ≥18 years old, received inpatient or outpatient services, and were tested for SARS-CoV-2 at 16 VHA medical centers across the U.S. from June 2020 through September 2022 [11,12]. The CDW is a centralized national repository of VHA EHR data used for research, quality improvement, and operational support [13]. For this analysis, we included EPIC^3^ participants who were enrolled in the context of inpatient care (inpatient cohort) or outpatient care (outpatient cohort). We excluded individuals who had no reverse transcription polymerase chain reaction (RT-PCR) test results for SARS-CoV-2 within 30 days prior to enrollment; self-reported a history of diabetes; or had zero days of follow-up. We also excluded individuals with evidence suggestive of diabetes prior to their index date based on laboratory values from plasma or serum (random glucose ≥200 mg/dL, fasting glucose ≥126 mg/dL, two-hour glucose from an oral glucose tolerance test ≥200 mg/dL) or whole blood (HbA1c ≥ 6.5%) [14], International Classification of Diseases and Related Health Problems, 10^th^ Revision (ICD-10) codes consistent with a diagnosis of diabetes, and receipt of glucose-lowering medications (**S1****–****S3 Tables**). Furthermore, all participants were required to have a laboratory test (serum or plasma glucose or HbA1c) that was not consistent with a diagnosis of diabetes in the 24 months prior to their index date. Fasting glucose was defined using tests drawn between 6:00 and 10:00 AM and concurrently with a low-density lipoprotein (LDL) test; all other glucose measurements were considered random. This resulted in a final analytic population of 1,212, of whom 826 were confirmed as SARS-CoV-2 positive and 386 SARS-CoV-2 negative at enrollment by RT-PCR (**Fig 1**). Of these, n = 161 were enrolled as inpatients, and n = 1,051 were enrolled as outpatients. The study was approved by the VA Central Institutional Review Board. Veterans or their legally authorized representative provided written informed consent for participation.

### Study variables

Trained study staff endeavored to collect questionnaires and biospecimens at enrollment and on days 3, 7, 14, 21, 28, as well as approximately months 3, 6, 12, 18, and 24, which were supplemented by EHR data.

We used RT-PCR tests recorded in the CDW to define SARS-CoV-2 exposure over time. Each participant contributed one or more SARS-CoV-2 episodes defined by qualifying RT-PCR tests. The time origin for each episode was the date of the SARS-CoV-2 test defining that episode. Participants began in a negative episode if they had no prior positive test and ≥1 confirmed negative test within 30 days prior to enrollment, or in a positive episode if they had ≥ 1 positive test within the 30 days prior to enrollment. Subsequent positive episodes were defined by a positive RT-PCR test occurring ≥90 days after the start of a prior episode. SARS-CoV-2 status was treated as time-varying, with person-time classified as unexposed until the start of a positive episode and exposed thereafter.

Patients were considered to have developed incident diabetes if they fulfilled any of the following criteria during follow-up: (1) two or more abnormal laboratory values from plasma or serum or whole blood as above [14]; or (2) inpatient or outpatient ICD-10 codes of E08–E13; or (3) receipt of an initial outpatient and one refill prescription of a glucose-lowering medication (alpha-glucosidase inhibitor, dipeptidyl peptidase-4 inhibitor, insulin, peroxisome proliferator–activated receptor gamma agonist, sulfonylurea, or amylin analogue). We did not include glucose-lowering medications with common non-diabetes indications in these criteria (i.e., metformin [used for prediabetes and PCOS], GLP-1 receptor agonists [obesity], SGLT2 inhibitors [heart failure or chronic kidney disease]). To avoid capturing transient hyperglycemia related to acute illness from SARS-CoV-2 or treatment with systemic corticosteroids, we excluded glucose values collected between day 0 and day 28 after enrollment for participants in both groups.

We collected data on demographic and clinical characteristics from responses to the modified Million Veteran Program (MVP) COVID-19 survey, which was administered to EPIC^3^ participants at enrollment [15], and from CDW records. Body mass index (BMI) was calculated based on height and weight extracted from the electronic health record. Never smokers were defined as those who had smoked <100 cigarettes in their lifetime, while former smokers reported smoking ≥100 cigarettes but were not currently smoking. The Charlson Comorbidity Index (CCI), a weighted summary measure of 17 chronic conditions used to quantify overall documented comorbidity burden [16], was calculated for each participant; missing CCI values were substituted using the participant’s baseline values, or imputed if a baseline CCI was not available.

### Statistical analyses

We examined distributions of covariates according to baseline and time-varying exposure status (SARS-CoV-2–positive vs. –negative and recruitment setting (inpatient and outpatient). We compared differences in post-enrollment clinical care-seeking behaviors by exposure status using Wilcoxon rank-sum tests, including differences in the number of days/year hospitalized, the number of unique clinic visit days/year, the number of laboratory test days/year, and the number of unique days/year during which a participant had a glucose or HbA1c test. We used extended Kaplan-Meier analysis to estimate diabetes-free survival stratified by time-varying SARS-CoV-2 status. We fit time-varying Cox proportional hazards models with a site-level frailty term to estimate hazard ratios (HR) for incident diabetes. SARS-CoV-2 status was treated as a time-varying covariate; participants who were SARS-CoV-2 negative at baseline contributed person-time to the SARS-CoV-2–negative group but began contributing to the positive group upon a positive RT-PCR test. Follow-up was censored at diagnosis of type 1 or other non–type 2 diabetes, death, withdrawal from the study, or last recorded VHA encounter, and participants contributed at most one incident diabetes event. We fit one unadjusted model (Crude) and three adjusted models. Models 1 and 2 adjusted for age, sex, race, smoking status, and CCI, plus number of unique laboratory test days in the year prior to enrollment (model 1 only) and number of unique glucose or HbA1c test days in the year prior to enrollment (model 2 only). Model 3 additionally adjusted for BMI and education (Model 1 + BMI and education). Given the small number of events, these adjustment sets were selected to balance parsimony with inclusion of covariates plausibly upstream of SARS-CoV-2 test positivity and diabetes risk, focusing on demographic characteristics, comorbidity burden, and prior health care utilization. To evaluate potential risk of outcome misclassification due to clinician overcoding of diabetes [17], we examined methods of diabetes detection (ICD-10 codes vs. laboratory tests) by exposure status. We used the ‘mice’ package in R to perform multiple imputation (m = 10, maxit = 20) of missing covariate values [18] using an imputation model including demographic characteristics, comorbidities, health care utilization measures, and diabetes outcome indicators. Overall, missingness was low for most covariates (sex 0.2%, BMI 1.8%, smoking 12.4%, race 14.1%, education 12.7%, rurality 13.4%), with some exceptions (e.g., income 37.6%). We checked Schoenfeld residuals to assess whether the proportional hazards assumption held for the Cox PH models. Significance level was set as two-sided alpha = 0.05 and all the analyses were conducted in R (Version 4.3.2).

## Results

Median age of participants was 51.6 years, and most (79.4%) were men. At baseline, participants with SARS-CoV-2 were younger compared to those without (50.1 vs. 57.2 years, respectively) had a higher income (18.2% vs. 15.3% with an annual income ≥ $100,000), were less likely to smoke (11.9% vs. 17.9% self-reported current smoker), and were more likely to be unvaccinated for SARS-CoV-2 (50.6% vs. 37.0% with no vaccinations). Participants with a positive test for SARS-CoV-2 also had fewer clinic visits (14.0 vs. 20.0), and glucose tests (1.0 vs. 2.0) in the year prior to enrollment; however, the number of laboratory tests and HbA1c tests in the year prior were similar (2.0 vs. 2.0, 0.7 vs. 0.7, respectively). HbA1c value in the two years prior to enrollment was also similar (5.5% vs. 5.5%). Among 386 participants who were SARS-CoV-2–negative at enrollment, 62 converted to positive during follow-up, with a median time to conversion of 298 days [IQR 146–479]. After accounting for time-varying SARS-CoV-2 infection status, 6.5% (54) of participants with a positive test for SARS-CoV-2 developed diabetes compared to 6.7% (26) of participants with a negative test over a median follow-up time of 4.3 and 4.1 person-years respectively; corresponding incidence rates were 16.1 and 21.2 cases per 1000 person-years (**Table 1**). Characteristics of participants by care setting (inpatient vs. outpatient) and SARS-CoV-2 test status (positive vs. negative) at enrollment are shown in S4 Table.

**Table 1:** Characteristics of EPIC^3^ participants who were free from diabetes at baseline stratified by SARS-CoV-2 status, n = 1,212.

	SARS-CoV-2 negative at enrollment^1^	SARS-CoV-2 positive at enrollment^1^	p-value	Weighted by SARS-CoV-2 negative person-years^2^	Weighted by SARS-CoV-2 positive person-years^2^
N, total participants/ total person-years	386	826		1414.6	3719.6
Age, years	57.2 (41.4, 67.0)	50.1 (37.8, 63.7)	<0.001	56.0 (41.0, 65.8)	50.1 (38.0, 63.6)
Sex			0.5		
Female	84 (21.8%)	163 (19.8%)		282.6 (23.1%)	684.3 (20.8%)
Male	302 (78.2%)	660 (80.2%)		941.8 (76.9%)	2599.7 (78.9%)
Race			0.081		
White	264 (68.4%)	573 (69.4%)		850.0 (69.4%)	2287.9 (69.5%)
Black/ African American	91 (23.6%)	155 (18.8%)		275.0 (22.5%)	617.0 (18.7%)
Other or Multiracial	22 (5.7%)	67 (8.1%)		72.9 (6.0%)	268.0 (8.1%)
Unknown	< 10	31 (3.8%)		--	121.1 (3.7%)
Highest education			>0.9		
High school or less	51 (13.2%)	103 (12.5%)		155.3 (12.7%)	424.2 (12.9%)
Above high school	292 (75.6%)	627 (75.9%)		950.9 (77.7%)	2540.0 (77.1%)
Unknown	43 (11.1%)	96 (11.6%)		118.3 (9.7%)	329.8 (10.0%)
Residence			0.13		
Highly rural	< 10	< 10		--	--
Rural	38 (9.8%)	117 (14.2%)		134.6 (11.0%)	427.7 (13.0%)
Urban	341 (88.3%)	699 (84.6%)		1070.7 (87.4%)	2433.1 (73.9%)
Unknown	< 10	< 10		--	--
Annual income			<0.001		
< $20,000	34 (8.8%)	39(4.7%)		86.6 (7.1%)	190.1 (5.8%)
$20,000-$49,999	86 (22.3%)	130 (15.7%)		275.3 (22.5%)	523.6 (15.9%)
$50,000-$99,999	71 (18.4%)	202 (24.5%)		220.5 (18.0%)	813.6 (24.7%)
≥ $100,000	59 (15.3%)	150 (18.2%)		199.9 (16.3%)	597.8 (18.1%)
Unknown	136 (35.2%)	305 (36.9%)		442.2 (36.1%)	1168.9 (35.5%)
Smoking status			0.036		
Never	149 (38.6%)	358 (43.3%)		486.6 (39.7%)	1459.4 (44.3%)
Former	123 (31.9%)	277 (33.5%)		397.5 (32.5%)	1110.3 (33.7%)
Current	69 (17.9%)	98 (11.9%)		215.8 (17.6%)	404.4 (12.3%)
Unknown	45 (11.7%)	93 (11.3%)		124.6 (10.2%)	319.9 (9.7%)
SARS-CoV-2 vaccination status^3^			<0.001		
No vaccination	143 (37.0%)	418 (50.6%)		422.2 (34.5%)	1590.1 (48.3%)
One dose	87 (22.5%)	109 (13.2%)		309.7 (25.3%)	430.1 (13.1%)
Complete dose	57 (14.8%)	159 (19.2%)		167.5 (13.7%)	629.8 (19.1%)
Complete dose plus booster	99 (25.6%)	140 (16.9%)		325.1 (26.5%)	644.1 (19.6%)
Charlson Comorbidity Index (CCI)	0.0 (0.0, 2.0)	0.0 (0.0, 1.0)	<0.001	0.0 (0.0, 1.0)	0.0 (0.0, 1.0)
Body Mass Index (BMI), kg/m^2^	29.3 (25.7, 33.1)	29.3 (25.8, 33.7)	0.4	29.7 (25.7, 33.2)	29.3 (25.9, 33.6)
SARS-CoV-2 positive episodes per person	0.0 (0.0, 0.0)	1.0 (1.0, 1.0)	<0.001	0.0 (0.0, 0.0)	1.0 (1.0, 1.0)
Most recent HbA1c result prior to enrollment^4^	5.5 (5.2, 5.8)	5.5 (5.2, 5.8)	0.4	5.5 (5.2, 5.8)	5.5 (5.2, 5.7)
Unknown, number of participants	218	551			
Days hospitalized^5^	0.0 (0.0, 1.0)	0.0 (0.0, 0.0)	0.039	0.0 (0.0, 1.6)	0.0 (0.0, 0.8)
Unique clinical visits, days^5^	20.0 (9.0, 32.0)	14.0 (7.0, 25.0)	<0.001	22.7 (13.5, 38.8)	16.4 (8.8, 28.7)
Unique lab tests (non-SARS-CoV-2), days^5^	2.0 (1.0, 5.0)	2.0 (1.0, 4.0)	<0.001	3.5 (2.0, 7.1)	2.7 (1.5, 4.9)
Unique glucose or HbA1c tests, days^5^	2.0 (1.0, 4.0)	1.0 (1.0, 3.0)	<0.001	2.6 (1.5, 4.6)	2.0 (1.1, 3.5)
Unique glucose tests, days^5^	2.0 (1.0, 3.0)	1.0 (1.0, 2.0)	<0.001	2.3 (1.4, 4.3)	1.7 (1.0, 3.0)
Unique HbA1c tests, days^5^	1.0 (0.0, 1.0)	1.0 (0.0, 1.0)	0.012	0.7 (0.4, 1.1)	0.7 (0.4, 1.1)
Follow-up time, person-years	4.0 (3.4, 4.6)	4.1 (3.6, 4.5)	0.12	—	—
Incident diabetes, cases during follow-up	—	—		26 (6.7%)	54 (6.5%)
Incident diabetes, cases per 1000 person-years	—	—		21.2	16.1

^1^Data are presented as median (Q1, Q3) for continuous variables or n (%) for categorical variables based on SARS-CoV-2 status at enrollment.

^2^Weighted values are presented as median (Q1, Q3) weighted by SARS-CoV-2 negative/positive person-years for continuous variables or negative/positive person-years (%) for categorical variables based on SARS-CoV-2 status at the beginning of each episode.

^3^as of one week prior to enrollment.

^4^within 180 days prior to enrollment.

^5^within one year prior to enrollment.

**Fig 2:**
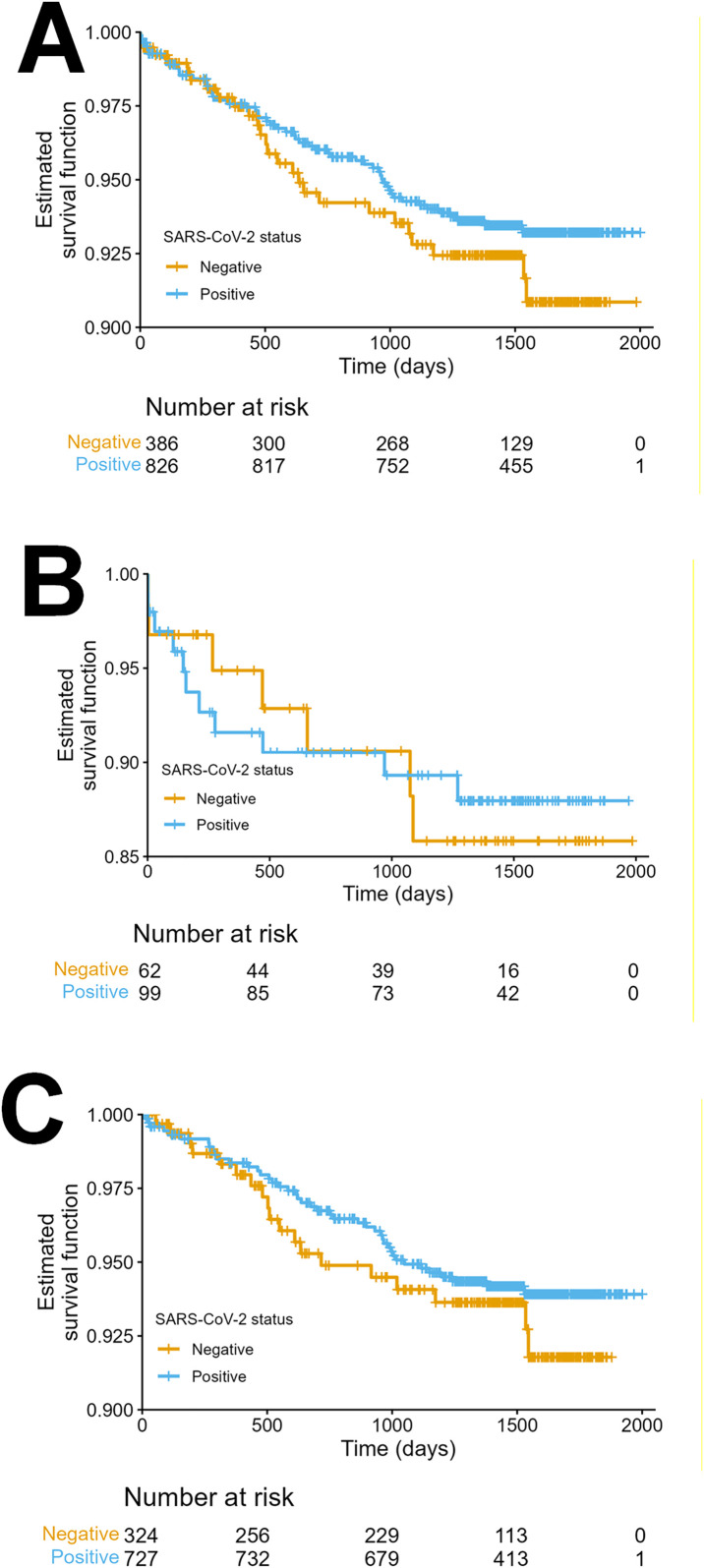
Extended Kaplan-Meier curves comparing the incidence of diabetes between EPIC^3^ participants. Overall and stratified by recruitment setting and time-varying SARS-CoV-2 status. (A) overall, (B) in participants recruited for the inpatient cohort, and (C) in participants recruited for the outpatient cohort.

Using a time-varying exposure to define SARS-CoV-2 status at each episode, compared to participants with a negative SARS-CoV-2 test, participants with a positive test had fewer days on which they had clinic visits (18.2 days/year vs. 25.4 days/year, p < 0.001), laboratory tests of any kind (3.1 days/year vs. 4.3 days/year, p < 0.001), and random or fasting glucose tests (1.8 days/year vs. 2.8 days/year, p < 0.001); however, the number of days/year on which they had HbA1c tests was not significantly different (0.7 days/year vs. 0.7 days/year, p = 0.094). In both positive and negative participants, most glucose tests (93.1% and 90.0%, respectively) and all HbA1c tests were ordered as part of routine clinical care and not at regular study follow-up visits. (**Table 2**). There were no significant differences in adjusted diabetes-free survival in the overall population or among populations of participants recruited as inpatient or outpatients (model 1 adjusted HR 0.86 [95% CI 0.52–1.39], 1.01 [95% CI 0.29–3.53], and 0.82 [95% CI 0.46–1.45], respectively; model 2 adjusted HR 0.87 [95% CI 0.54–1.43], 1.01 [95% CI 0.29–3.48], and 0.83 [95% CI 0.47–1.47], respectively; model 3 adjusted HR 0.82 [95% CI 0.51–1.35], 1.03 [95% CI 0.27–3.96], and 0.79 [95% CI 0.44–1.39], respectively; **Table 3**, **Fig 2**). Among participants who were classified as having incident diabetes, the method of diabetes detection was based on diagnosis codes in 17 (32%) of positive and 9 (35%) of negative participants (**Table 4**).

**Table 2:** Patterns of post-enrollment clinical surveillance during SARS-CoV-2 episodes for EPIC^3^ participants with positive and negative tests for SARS-CoV-2, overall and stratified by hospitalization status at the beginning of each episode.

	Overall			Inpatient			Outpatient		
	SARS-CoV-2 positive n = 982	SARS-CoV-2 negative n = 386	p- value^2^	SARS-CoV-2 positive n = 113	SARS-CoV-2 negative n = 62	p- value^2^	SARS-CoV-2 positive n = 869	SARS-CoV-2 negative n = 324	p- value^2^
Days hospitalized^1^	0.0 (0.0, 0.9)	0.0 (0.0, 2.1)	<0.001	5.3 (1.9, 19.4)	10.1 (1.8, 20.5)	0.552	0.0 (0.0, 0.0)	0.0 (0.0, 0.5)	0.011
Unique clinical visit-days^1^	18.2 (9.7, 31.5)	25.4 (14.5, 44.6)	<0.001	13.3 (0.8, 26.8)	19.8 (1.3, 36.0)	0.138	9.2 (0.2, 17.3)	14.0 (1.0, 24.2)	<0.001
Unique laboratory test-days^1^	3.1 (1.7, 7.0)	4.3 (2.2, 12.2)	<0.001	7.0 (3.5, 21.1)	11.5 (5.4, 21.9)	0.073	2.9 (1.6, 6.3)	3.7 (2.1, 9.9)	<0.001
Unique glucose or HbA1c test-days^1^	2.2 (1.2, 4.7)	3.2 (1.6, 8.0)	<0.001	4.8 (2.7, 11.3)	7.9 (4.1, 15.4)	0.067	2.0 (1.1, 4.0)	2.8 (1.5, 5.8)	<0.001
Unique glucose test-days^1^	1.8 (1.0, 3.3)	2.8 (1.4, 5.4)	<0.001	3.7 (2.4, 7.9)	6.5 (3.9, 13.8)	0.015	1.6 (0.9, 2.9)	2.5 (1.3, 4.4)	<0.001
Unique HbA1c test-days^1^	0.7 (0.4, 1.1)	0.7 (0.2, 1.1)	0.094	0.7 (0.3, 1.1)	0.6 (0.2, 1.2)	0.659	0.7 (0.4, 1.1)	0.7 (0.2, 1.1)	0.108
Percent of glucose and HbA1c tests ordered by EPIC^3^ (%)	6.9	10.0	—	9.5	7.0	—	6.2	11.1	—

^1^median (Q1, Q3) days per 365 days.

^2^p-values from Wilcoxon rank-sum tests.

**Table 3:** Risk of incident diabetes comparing episodes among EPIC^3^ participants with positive vs. negative tests for SARS-CoV-2.

Population	Model	HR	p-value^1^	95% CI
**Overall**	Crude	0.76	0.27	0.47–1.24
	Model 1	0.86	0.53	0.52–1.39
	Model 2	0.87	0.60	0.54–1.43
	Model 3	0.82	0.44	0.51–1.35
**Inpatient**	Crude	0.91	0.85	0.33–2.55
	Model 1	1.01	0.98	0.29–3.53
	Model 2	1.01	0.99	0.29–3.48
	Model 3	1.03	0.96	0.27–3.96
**Outpatient**	Crude	0.77	0.35	0.44–1.34
	Model 1	0.82	0.48	0.46–1.45
	Model 2	0.83	0.51	0.47–1.47
	Model 3	0.79	0.40	0.44–1.39

Abbreviations: CCI (Charlson comorbidity index), CI (confidence interval), HR (hazard ratio).

Model 1 adjusted for age, sex, race, smoking status, CCI, and number of unique laboratory test days in the year prior to enrollment.

Model 2 adjusted for age, sex, race, smoking status, CCI, and number of unique glucose or HbA1c test days in the year prior to enrollment.

Model 3 adjusted for age, sex, race, smoking status, BMI, education level, CCI, and number of unique laboratory test days in the year prior to enrollment.

^1^Assessed using Wald tests.

**Table 4:** Methods of diabetes detection among EPIC^3^ participants with positive and negative tests for SARS-CoV-2 at each episode, overall and stratified by inpatient vs. outpatient at the beginning of the SARS-CoV-2 episode.

	SARS-CoV-2 status at beginning of episode	≥2 ICD-10 codes	≥2 HbA1c tests	≥2 glucose tests	1 glucose test and 1 HbA1c test
Overall	Positive, n = 54	17 (32%)	14 (26%)	14 (26%)	9 (17%)
	Negative, n = 26	9 (35%)	7 (27%)	6 (23%)	4 (16%)
Inpatient at episode	Positive, n = 11	3 (27%)	3 (27%)	3 (27%)	2 (18%)
	Negative, n = 7	4 (57%)	2 (18%)	0 (0%)	1 (9%)
Outpatient at episode	Positive, n = 43	14 (33%)	11 (26%)	11 (26%)	7 (16%)
	Negative, n = 19	5 (26%)	5 (26%)	6 (32%)	3 (16%)

Abbreviations: ICD-10 (International Classification of Diseases, 10th Revision).

Earliest ascertainment by date, or by the order shown here if on same date.

## Discussion

In this prospective longitudinal cohort of Veterans with and without recent SARS-CoV-2 infection, Veterans with SARS-CoV-2 who enrolled in the study were younger, had higher income, smoked less, and were less likely to be vaccinated. While we observed differences in metrics of healthcare utilization between the SARS-CoV-2-positive and -negative groups, rates of HbA1c testing were similar. Compared to the general population of U.S. adults (in whom the incidence of diagnosed diabetes was about 6.4 per 1000 person-years during a similar time period [19]), incidence of diabetes was high in the study population (16.1 and 21.2 cases per 1,000 person-years among SARS-CoV-2 positive and negative participants, respectively) after accounting for time-varying SARS-CoV-2 infection status. This corroborates earlier findings that demonstrate that, even when matched for age and comorbidities, the incidence of diabetes is higher among Veterans compared to the general population [20–23]. The risk of incident diabetes in models adjusted for clinical characteristics and laboratory testing in the year prior to enrollment was similar among those with and without SARS-CoV-2 infection.

To our knowledge, this study is the first to compare diabetes incidence in individuals with positive and negative RT-PCR tests for SARS-CoV-2 at baseline in a prospective design. Consistent with the current findings, in a 2025 retrospective analysis in a propensity score–matched cohort of U.S. active-duty service members and their families identified through the Military Health System Data Repository, SARS-CoV-2 infection was not associated with incident diabetes (HR 0.95; 99% CI: 0.75, 1.21) [24]. Whereas these two studies found no evidence of an association of SARS-CoV-2 infection with incident diabetes, large retrospective cohort studies and meta-analyses (including a very recent meta-analysis) [25] have reported an elevated risk of new-onset diabetes following COVID-19 compared with non-infected controls. In those studies, associations are typically strongest among individuals with more severe disease and during the time period closest to infection. In pooled estimates from meta-analyses of retrospective studies, SARS-CoV-2 was consistently associated with a 50–80% higher risk [1–6], which differs substantially from our findings. Reasons for these conflicting results could include differences in data collection, missing data, diabetes definitions used, selection bias, surveillance bias, confounding control, or insufficient sample size. For example, because diabetes incidence peaks in the 55–64 year old age group, the older age of the SARS-CoV-2-negative participants in our study may contribute to the higher diabetes incidence in that group [26]. Likewise, the relatively low number of participants with severe SARS-CoV-2 (using hospitalization for SARS-CoV-2 as a surrogate for severity) may account for the lack of an association between SARS-CoV-2 infection and diabetes incidence because prior studies have shown that those with severe disease are at a higher risk than those with mild disease for subsequently developing diabetes.

In the acute (i.e., inpatient) setting, SARS-CoV-2 infection/COVID is associated with adverse short-term metabolic outcomes [27], including higher glucose levels and greater insulin requirements. The longer-term impacts of SARS-CoV-2 on metabolic health are, however, less clear. We previously observed that Veterans with incident diabetes following SARS-CoV-2 infection exhibited HbA1c levels comparable to those with incident diabetes without documented SARS-CoV-2 exposure [28]. However, individuals with a history of SARS-CoV-2 infection were more frequently initiated on insulin therapy in the subsequent 120 days. These observations may be attributable to several underlying mechanisms. One possibility is that diabetes occurring after COVID requires more intensive therapeutic intervention to achieve equivalent glycemic control, potentially due to virus-induced pathophysiological changes such as increased insulin resistance or pancreatic β-cell dysfunction [29]. Alternatively, the higher rate of insulin prescriptions in this cohort may reflect increased healthcare utilization or heightened clinical vigilance following SARS-CoV-2 infection.

### Limitations

Our study has several strengths. First, the analysis was conducted in the context of a prospective observational cohort study that had regular follow-up as part of its longitudinal design. We also adjusted for health care utilization in the year prior to enrollment and excluded glucose tests in the initial 28 days after enrollment, all strategies that may mitigate bias due to differences in surveillance. The integration of VHA medical records data going back to 1999 allowed us to exclude individuals with prevalent diabetes at baseline. Further, we required confirmation of non-diabetic status with a recent laboratory test for diabetes prior to enrollment. These strategies are expected to reduce the possibility that individuals with prevalent diabetes were inappropriately counted as incident diabetes cases. We also acknowledge several important limitations. First, although EPIC^3^ participants received regular study follow-up for 24 months, this follow-up did not include longitudinal fasting glucose or HbA1c testing. Most of the relevant laboratory tests were obtained as part of regular clinical care, including as part of diabetes screening or other aspects of medical care. Given that there was more random glucose testing in the SARS-CoV-2 negative group, such differences could contribute to higher diabetes ascertainment in that group. We observed similar HbA1c testing rates, which reflects active clinician-initiated diabetes screening, though differences in other testing patterns and clinical encounters could still influence detection. We did not have baseline HbA1c measurement on all participants, which limited our ability to adjust for baseline glycemia. Residual confounding may remain from factors we could not fully adjust for (e.g., baseline glycemia) as well as from imperfect measurement or categorization of covariates we included (e.g., age). While residual confounding can bias estimates in either direction, the older age distribution in the SARS-CoV-2–negative group could bias the association downward (toward a lower apparent risk among those infected), given the known positive association of age with diabetes risk. Indications for SARS-CoV-2 testing and for hospitalization were not available, raising the possibility of selection differences between -positive and -negative participants, particularly in the inpatient subgroup. Requiring a recent laboratory test that excluded prevalent diabetes increased internal validity but may have selected for individuals more engaged in care.

We had limited statistical power to detect statistically significant differences between groups; this led to large confidence intervals around our estimates which hinders interpretation of our findings. The small number of incident diabetes events limited model stability and increased the risk of overfitting. There was also inadequate power to examine the roles of vaccination or receipt of systemic corticosteroid therapy in the development of diabetes (e.g., as potential effect modifiers, or, in the case of corticosteroid therapy, as a potential mediator). Study enrollment and follow-up occurred across multiple phases of the pandemic, including periods dominated by ancestral SARS-CoV-2 strains, followed by Alpha, Delta, and Omicron variants (BA.1–BA.5), which may have distinct metabolic effects [30] that we were unable to evaluate due to sample size. Finally, because of the rigorous inclusion criteria we used to strengthen internal validity, results may not be generalizable to, e.g., Veteran populations who do not receive regular medical care and laboratory testing. Higher baseline healthcare engagement in the analytic cohort is also expected to increase rates of diabetes detection.

### Future directions

To definitively determine the association of SARS-CoV-2 infection with incident diabetes, large-scale prospective studies with standardized glycemic assessment, longer follow-up, and careful phenotyping by infection severity and metabolic risk are needed to clarify the contribution of SARS-CoV-2 to diabetes risk in specific subgroups.

## Conclusions

In this prospective longitudinal cohort study of U.S. Veterans with and without recent SARS-CoV-2 infection, we observed some differences in post-enrollment care-seeking behaviors comparing SARS-CoV-2–positive and –negative participants, although rates of HbA1c testing were similar. Incident diabetes was relatively common, with rates of 16.1 and 21.2 cases per 1,000 person-years among SARS-CoV-2 positive and negative participants, respectively. We found no evidence of higher risk of incident diabetes after SARS-CoV-2 infection within the observed follow-up interval, although modest associations cannot be excluded given the sample size and event count. The findings do not support changing current clinical practice, which advises routine screening beginning at age 35, and earlier or more frequent testing is recommended in those with overweight/obesity or additional risk factors, while highlighting the need for longer structured follow-up with repeated structured glycemic assessments to identify subgroups that may benefit from targeted screening or surveillance. Future studies comparing SARS-CoV-2–positive and –negative participants must consider how differences in surveillance may impact outcome ascertainment, especially for conditions that are detected by laboratory testing.

## Supporting information

S1 Table Definitions used to classify diabetes status at baseline. (DOCX)

S2 Table ICD-10 codes used for diabetes definitions. (DOCX)

S3 Table Glucose-lowering medications used in diabetes definitions. (DOCX)

S4 Table Characteristics of participants by care setting (inpatient vs. outpatient) and SARS-CoV-2 test status (positive vs. negative) at enrollment. (DOCX)
