# Supplementary material for: Association of SARS-CoV-2 infection with incident diabetes among U.S. Veterans in a prospective longitudinal cohort
Source: PLoS One. 2026 Jun 26;21(6):e0351992. doi: 10.1371/journal.pone.0351992 (PMC13308785; doi:10.1371/journal.pone.0351992)
Supplement: S1 Table — (DOCX) [file pone.0351992.s001.docx]

| **Supplemental Table 1.** Definitions used to classify diabetes status at baseline | |
| --- | --- |
| **Criterion** | **Category** |
| Presence of ≥1 ICD-9-CM or ICD-10 code for diabetes, other (E08.x, E09.x, E13.x) | Confirmed: Other diabetes |
| Presence of ≥1 ICD-9-CM or ICD-10 code for T1D (E10.x) | Confirmed: T1D |
| *If participants fulfill any of the following criteria:* | Confirmed: T2D |
| Presence of ≥1 ICD-10 code for T2D (E11.x) while an inpatient, or ≥2 ICD-10 codes for T2D while an outpatient, or ≥1 ICD-10 code for T2D while and outpatient and ≥1 ICD-10 code for T2D while an inpatient |  |
| HbA1c ≥ 6.5% 2 or more times, or |  |
| Random glucose ≥ 200 mg/dL 2 or more times, or |  |
| Fasting glucose^a^ ≥ 126 mg/dL 2 or more times, or |  |
| 2 h OGTT ≥ 200 mg/dL 2 or more times, or |  |
| Random glucose ≥ 200 mg/dL for at least once and HbA1c ≥ 6.5% for at least once, or |  |
| Fasting glucose^a^ ≥ 126 mg/dL at least once and HbA1c ≥ 6.5% at least once, or |  |
| Receipt of glucose-lowering medication ≥2 times |  |
| *If participants fulfill any of the following criteria:* | Confirmed to be prediabetes, suspected to be T2D |
| HbA1c between 5.7% and 6.5% at least once, or |  |
| Fasting glucose^a^ between 100 and 125 mg/dL at least once, or |  |
| 2 h OGTT between 140 and 200 mg/dL at least once, or |  |
| Presence of ICD code for prediabetes ≥1 time |  |
| *AND* |  |
| HbA1c ≥ 6.5% once, or |  |
| Fasting glucose^a^ ≥ 126 mg/dL once, or |  |
| Random glucose ≥ 200 mg/dL once, or |  |
| 2 h OGTT ≥ 200 mg/dL once |  |
| *If participants fulfill any of the following criteria:* | Confirmed: Prediabetes |
| HbA1c between 5.7% and 6.5% at least once, or |  |
| Fasting glucose between 100 and 125 mg/dL at least once, or |  |
| 2 h OGTT between 140 and 200 mg/dL at least once, or |  |
| Presence of ICD code for prediabetes ≥1 time |  |
| *AND no any other indication for T2D or suspected T2D* |  |
| *If participants fulfill any of the following criteria:* | Suspected: T2D |
| HbA1c ≥ 6.5% once, or |  |
| Fasting glucose^a^ ≥ 126 mg/dL once, or |  |
| Random glucose ≥ 200 mg/dL once, or |  |
| 2 h OGTT ≥ 200 mg/dL once |  |
| *If participants do not belong to any of the situations mentioned above, meanwhile fulfill any of the following indication:* | Confirmed: No diabetes |
| HbA1c < 5.7 mg/dL, or |  |
| Random glucose < 200 mg/dL, or |  |
| Fasting glucose^a^ < 100 mg/dL, or |  |
| OGTT < 140 mg/dL |  |
| *If participants do not fulfill any criteria above and lacking laboratories that meet criteria for Confirmed: No diabetes* | Suspected: No diabetes |
| Abbreviations: T1D: Type 1 Diabetes; T2D: Type 2 Diabetes; HbA1c: Hemoglobin A1c test; random glucose: Random glucose test; fasting glucose: Fasting glucose test; OGTT: 2-hour oral glucose tolerance test. | |
| ^a^Categorization of fasting glucose to a glucose that was time-matched on an LDL-c blood draw in a clinic setting between the hours of 6 am-10am. | |
