# Supplementary material for: Association of SARS-CoV-2 infection with incident diabetes among U.S. Veterans in a prospective longitudinal cohort
Source: PLoS One. 2026 Jun 26;21(6):e0351992. doi: 10.1371/journal.pone.0351992 (PMC13308785; doi:10.1371/journal.pone.0351992)
Supplement: S2 Table — (DOCX) [file pone.0351992.s002.docx]

| **Supplemental Table 2. ICD-10 codes used for diabetes definitions** | |
| --- | --- |
| **Classification** | **ICD-10 Codes** |
| **T1D** | E10.10, E10.11, E10.21, E10.22, E10.29, E10.311, E10.319, E10.321, E10.3211, E10.3212, E10.3213, E10.3219, E10.329, E10.3291, E10.3292, E10.3293, E10.3299, E10.331, E10.3311-E10.3313, E10.3319, E10.339, E10.3391-E10.3393, E10.3399, E10.341, E10.3411-E10.3413, E10.3419, E10.349, E10.3491-E10.3493, E10.3499, E10.351, E10.3511-E10.3513, E10.3519, E10.3521-E10.3523, E10.3529, E10.3531-E10.3533, E10.3539, E10.3541-E10.3543, E10.3549, E10.3551-E10.3553, E10.3559, E10.359, E10.3591-E10.3593, E10.3599, E10.36, E10.37X1-E10.37X3, E10.37X9, E10.39-E10.44, E10.49, E10.51, E10.52, E10.59, E10.610, E10.618, E10.620, E10.621, E10.622, E10.628, E10.638, E10.638, E10.641, E10.649, E10.65, E10.69, E10.8, E10.9 |
| **T2D** | E11.00, E11.01, E11.10, E11.11, E11.21, E11.22, E11.29, E11.311, E11.319, E11.321, E11.3211, E11.3212, E11.3213, E11.3219, E11.329, E11.3291, E11.3292, E11.3293, E11.3299, E11.331, E11.3311, E11.3312, E11.3313, E11.3319, E11.339, E11.3391, E11.3392, E11.3393, E11.3399, E11.341, E11.3411, E11.3412, E11.3413, E11.3419, E11.349, E11.3491, E11.3492, E11.3493, E11.3499, E11.351, E11.3511, E11.3512, E11.3513, E11.3519, E11.3521, E11.3522, E11.3523, E11.3529, E11.3531, E11.3532, E11.3533, E11.3539, E11.3541, E11.3542, E11.3543, E11.3549, E11.3551, E11.3552, E11.3553, E11.3559, E11.359, E11.3591, E11.3592, E11.3593, E11.3599, E11.36, E11.37X1, E11.37X2, E11.37X3, E11.37X9, E11.39-E11.44, E11.49, E11.51, E11.52, E11.59, E11.610, E11.618, E11.620, E11.621, E11.622, E11.628, E11.630, E11.638, E11.641, E11.649, E11.65, E11.69, E11.8, E11.9 |
| **Diabetes, other** | E08.00, E08.01, E08.10, E08.11, E08.21, E08.22, E08.29, E08.311, E08.319, E08.321, E08.3211-E08.3213, E08.3219, E08.329, E08.3291-E08.3293, E08.3299, E08.331, E08.3311-E08.3313, E08.3319, E08.339, E08.3391-E08.3393, E08.3399, E08.341, E08.3411-E08.3413, E08.3419, E08.349, E08.3491-E08.3493, E08.3499, E08.351, E08.3511-E08.3513, E08.3519, E08.3521-E08.3523, E08.3529, E08.3531-E08.3533, E08.3839, E08.3541-E08.3543, E08.3549, E08.3551-E08.3553, E08.3559, E08.359, E08.3591-E08.3593, E08.3599, E08.36, E08.37X1-E08.37X3, E08.37X9, E08.39-E08.44, E08.49, E08.51,E08.52, E08.59, E08.610, E08.618, E08.620-E08.622, E08.628, E08.630, E08.638, E08.641, E08.649, E08.65, E08.69, E08.8, E08.9, E09.00, E09.01, E09.10, E09.11, E09.21, E09.22, E09.29, E09.311, E09.319, E09.321, E09.3211-E09.3213, E09.3219, E09.329, E09.3291-E09.3293, E09.3299, E09.331, E09.3311-E09.3313, E09.3319, E09.339, E09.3391-E09.3393, E09.3399, E09.341, E09.3411-E09.3413, E09.3419, E09.349, E09.3492, E09.3493, E09.3499, E09.351, E09.3511-E09.3513, E09.3519, E09.3521, E09.3522, E09.3532, E09.3533, E09.3541-E09.3543, E09.3551-E09.3553, E09.3559, E09.359, E09.3591-E09.3593, E09.3599, E09.36, E09.37X1-E09.37X3, E09.39-E09.44, E09.49, E09.51, E09.52, E09.610, E09.618, E09.620-E09.622, E09.628, E09.630, E09.638, E09.641, E09.649, E09.65, E09.69, E09.8, E09.9, E13.00, E13.01, E13.10, E13.11, E13.21, E13.22, E13.29, E13.311, E13.319, E13.321, E13.3211-E13.3213, E13.3219, E13.329, E13.3291-E13.3293, E13.3299, E13.331, E13.3311-E13.3313, E13.3319, E13.339, E13.3391-E13.3393, E13.3399, E13.341, E13.3411-E13.3413, E13.3419, E13.349, E13.3491-E13.3493, E13.3499, E13.351, E13.3511-E13.3513, E13.3519, E13.3521-E13.3523, E13.3529, E13.3531-E13.3533, E13.3539, E13.3541-E13.3543, E13.3549, E13.3551-E13.3553, E13.3559, E13.359, E13.3591-E13.3593, E13.3599, E13.36, E13.37X1-E13.37X3, E13.37X9, E13.39-E13.44, E13.49, E13.51, E13.52, E13.59, E13.610, E13.618, E13.620-E13.622, E13.628, E13.630, E13.638, E13.641, E13.649, E13.65, E13.69, E13.8, E13.9 |
| Abbreviations: T1D: Type 1 Diabetes; T2D: Type 2 Diabetes; ICD-10 codes: International Classification of Diseases and Related Health Problems, 10th Revision codes | |
