# Supplementary material for: Association of SARS-CoV-2 infection with incident diabetes among U.S. Veterans in a prospective longitudinal cohort
Source: PLoS One. 2026 Jun 26;21(6):e0351992. doi: 10.1371/journal.pone.0351992 (PMC13308785; doi:10.1371/journal.pone.0351992)
Supplement: S3 Table — (DOCX) [file pone.0351992.s003.docx]

| **Supplemental Table 3. Glucose-lowering medications used in diabetes definitions** | |
| --- | --- |
| **Category** | **Drug names** |
| AGI | Acarbose, Miglitol |
| DPP-4 inhibitor | Alogliptin, Linagliptin, Saxagliptin, Sitagliptin |
| Insulin | Insulin, Fast-acting insulin (Apidra, Insulin Aspart), Insulin analog (Degludec), Short-acting insulin secretagogue (Nateglinide), Insulin secretagogue ( Repaglinide) |
| PPAR-gamma agonist | Arhalofenate (Partial-gamma agonist), Pioglitazone, Rosaglitazone |
| Sulfonylurea | Glimepiride, Glipizide, Glyburide, Tolazamide, Tolbutamide |
| Amlyn analogue | Pramlintide |
| Abbreviations: AGI: alpha-glucosidase inhibitors; DPP-4 inhibitor: dipeptidyl peptidase-4 inhibitor; PPAR-gamma agonist: peroxisome proliferator-activated receptor-gamma agonist | |
