# Supplementary material for: Association of SARS-CoV-2 infection with incident diabetes among U.S. Veterans in a prospective longitudinal cohort
Source: PLoS One. 2026 Jun 26;21(6):e0351992. doi: 10.1371/journal.pone.0351992 (PMC13308785; doi:10.1371/journal.pone.0351992)
Supplement: S4 Table — (DOCX) [file pone.0351992.s004.docx]

| **Supplemental Table 4.** Characteristics of participants by care setting (inpatient vs. outpatient) and SARS-CoV-2 test status (positive vs. negative) at enrollment, n= 1,212 | | | | | | |
| --- | --- | --- | --- | --- | --- | --- |
|  | **Inpatient at enrollment** | | | **Outpatient at enrollment** | | |
|  | **SARS-CoV-2 negative at enrollment** | **SARS-CoV-2 positive at enrollment** | **p-value** | **SARS-CoV-2 negative at enrollment** | **SARS-CoV-2 positive at enrollment** | **p-value** |
| N, participants | 62 | 99 |  | 324 | 727 |  |
| Age, years | 64.7 (57.1, 69.5) | 64.7 (52.0, 73.4) | 0.6 | 54.5 (38.9, 65.4) | 47.3 (37.3, 60.7) | < 0.001 |
| Sex |  |  | 0.4 |  |  | 0.2 |
| Female | < 10 | 11 (11.1%) |  | 80 (24.7%) | 152 (20.9%) |  |
| Male | 58 (93.5%) | 88 (88.9%) |  | 244 (75.3%) | 572 (78.7%) |  |
| Race |  |  | >0.9 |  |  | 0.13 |
| White | 37 (59.7%) | 59 (59.6%) |  | 227 (70.1%) | 514 (70.7%) |  |
| Black / African American | 21 (33.9%) | 31 (31.3%) |  | 70 (21.6%) | 124 (17.1%) |  |
| Other or Multiracial | < 10 | < 10 |  | 20 (6.2%) | 63 (8.7%) |  |
| Unknown | < 10 | < 10 |  | < 10 | 63 (8.7%) |  |
| Highest education |  |  | 0.3 |  |  | >0.9 |
| High school or less | 15 (24.2%) | 24 (24.2%) |  | 36 (11.1%) | 79 (10.9%) |  |
| Above high school | 32 (51.6%) | 40 (40.4%) |  | 260 (80.2%) | 587 (80.7%) |  |
| Unknown | 15 (24.2%) | 35 (35.4%) |  | 28 (8.6%) | 61 (8.4%) |  |
| Residence |  |  | 0.5 |  |  | 0.012 |
| Highly rural | -- | -- |  | < 10 | < 10 |  |
| Rural | 11 (17.7%) | 13 (13.1%) |  | 27 (8.3%) | 104 (14.3%) |  |
| Urban | 51 (82.3%) | 84 (84.8%) |  | 290 (89.5%) | 615 (84.6%) |  |
| Unknown | < 10 | < 10 |  | < 10 | < 10 |  |
| Annual income |  |  | 0.4 |  |  | 0.003 |
| <$20,000 | < 10 | < 10 |  | 28 (8.6%) | 33 (4.5%) |  |
| $20,000-$49,999 | 11 (17.7%) | 10 (10.1%) |  | 75 (23.1%) | 120 (16.5%) |  |
| $50,000-$99,999 | < 10 | 16 (16.2%) |  | 63 (19.4%) | 186 (25.6%) |  |
| ≥$100,000 | < 10 | < 10 |  | 57 (17.6%) | 142 (19.5%) |  |
| Unknown | 35 (56.5%) | 59 (59.6%) |  | 101 (31.2%) | 246 (33.8%) |  |
| Smoking status |  |  | 0.035 |  |  | 0.3 |
| Never | 10 (16.1%) | 27 (27.3%) |  | 139 (42.9%) | 331 (45.5%) |  |
| Former | 20 (32.3%) | 29 (29.3%) |  | 103 (31.8%) | 248 (34.1%) |  |
| Current | 15 (24.2%) | < 10 |  | 54 (16.7%) | 89 (12.2%) |  |
| Unknown | 17 (27.4%) | 34 (34.3%) |  | 28 (8.6%) | 59 (8.1%) |  |
| SARS-CoV-2 vaccination status^1^ |  |  | 0.6 |  |  | < 0.001 |
| No vaccination | 33 (53.2%) | 60 (60.6%) |  | 110 (34.0%) | 358 (49.2%) |  |
| One dose | 10 (16.1%) | 10 (10.1%) |  | 77 (23.8%) | 99 (13.6%) |  |
| Complete dose | < 10 | 13 (13.1%) |  | 50 (15.4%) | 146 (20.1%) |  |
| Complete dose plus booster | 12 (19.4%) | 16 (16.2%) |  | 87 (26.9%) | 124 (17.1%) |  |
| Charlson Comorbidity Index (CCI) | 2.0 (1.0, 3.0) | 1.0 (0.0, 3.0) | 0.2 | 0.0 (0.0, 1.0) | 0.0 (0.0, 1.0) | 0.002 |
| Body Mass Index (BMI), kg/m^2^ | 28.5 (23.2, 33.5) | 27.8 (24.9, 32.2) | >0.9 | 29.5 (26.0, 33.0) | 29.4 (26.0, 33.7) | 0.5 |
| SARS-CoV-2 positive episodes per person | 0.0 (0.0, 0.0) | 1.0 (1.0, 1.0) | < 0.001 | 0.0 (0.0, 0.0) | 1.0 (1.0, 1.0) | <0.001 |
| Most recent HbA1c result prior to enrollment^3^ | 5.8 (5.5, 6.0) | 5.6 (5.4, 5.8) | 0.3 | 5.5 (5.2, 5.7) | 5.4 (5.2, 5.8) | 0.8 |
| Unknown, number of participants | 31 | 69 |  | 187 | 482 |  |
| Days hospitalized^2^ | 0.0 (0.0, 1.0) | 0.0 (0.0, 1.0) | 0.8 | 0.0 (0.0, 0.0) | 0.0 (0.0, 0.0) | 0.051 |
| Unique clinical visits, days^2^ | 20.0 (6.0, 31.0) | 16.0 (5.0, 29.0) | 0.4 | 20.0 (9.0, 33.0) | 14.0 (7.0, 25.0) | < 0.001 |
| Unique lab tests (non-SARS-CoV-2), days^2^ | 3.0 (1.0, 6.0) | 2.0 (1.0, 6.0) | 0.2 | 2.0 (1.0, 5.0) | 2.0 (1.0, 4.0) | < 0.001 |
| Unique glucose or HbA1c tests, days^2^ | 3.0 (1.0, 7.0) | 2.0 (1.0, 5.0) | 0.059 | 2.0 (1.0, 4.0) | 1.0 (1.0, 3.0) | < 0.001 |
| Unique glucose tests, days^2^ | 2.0 (1.0, 5.0) | 2.0 (1.0, 4.0) | 0.15 | 1.0 (1.0, 3.0) | 1.0 (1.0, 2.0) | < 0.001 |
| Unique HbA1c tests, days^2^ | 1.0 (0.0, 1.0) | 1.0 (0.0, 1.0) | 0.048 | 1.0 (0.0, 1.0) | 1.0 (0.0, 1.0) | 0.061 |
| Follow-up time, person-years | 3.8 (2.8, 4.7) | 3.9 (2.0, 4.4) | 0.6 | 4.0 (3.5, 4.5) | 4.1 (3.6, 4.5) | 0.089 |
| Incident diabetes, cases during follow-up | 7 (11.3%) | 11 (11.1%) | >0.9 | 23 (7.1%) | 39 (5.4%) | 0.3 |
| Incident diabetes, cases per 1000 person-years | 33 (53.3%) | 3.3 (3.3%) |  | 19 (5.9%) | 11.64 (1.6%) |  |
| Data are presented as median (Q1, Q3) for continuous variables or n (%) for categorical variables.  ^1^ as of one week prior to enrollment  ^2^ within one year prior to enrollment  ^3^ within 180 days prior to enrollment | | | | | | |
