# Supplementary material for: Association of SARS-CoV-2 infection with incident diabetes among U.S. Veterans in a prospective longitudinal cohort
Source: PLoS One. 2026 Jun 26;21(6):e0351992. doi: 10.1371/journal.pone.0351992 (PMC13308785; doi:10.1371/journal.pone.0351992)
Supplement: Attachment — Submitted filename: response to reviewers_050426.docx [file pone.0351992.s006.docx]

4 May 2026

Dear editors:

Thank you for your thoughtful review and for the opportunity to revise this manuscript. We respond to the reviewers’ comments point-by-point below.

Great gratitude,

Luke

Pandora Lucrezia “Luke” Wander, MD, MS, FACP

Associate Professor of Medicine

Adjunct Associate Professor of Epidemiology & Public Health Genetics

University of Washington

Staff Physician

Veterans Affairs Puget Sound Health Care System

Journal Requirements:

When submitting your revision, we need you to address these additional requirements.

1. Please ensure that your manuscript meets PLOS ONE's style requirements, including those for file naming. The PLOS ONE style templates can be found at

[https://journals.plos.org/plosone/s/file?id=wjVg/PLOSOne_formatting_sample_main_body.pdf](https://urldefense.com/v3/__https:/journals.plos.org/plosone/s/file?id=wjVg*PLOSOne_formatting_sample_main_body.pdf__;Lw!!K-Hz7m0Vt54!ii_Um56GhKhrmcj_W4aJ-LsGIGze2C0w6bYSCYyzbg0UzYwEY7L54U4Cal71Gk-PGdY4vGb1fLB38Vs$) and

[https://journals.plos.org/plosone/s/file?id=ba62/PLOSOne_formatting_sample_title_authors_affiliations.pdf](https://urldefense.com/v3/__https:/track.editorialmanager.com/CL0/https:*2F*2Fjournals.plos.org*2Fplosone*2Fs*2Ffile*3Fid=ba62*2FPLOSOne_formatting_sample_title_authors_affiliations.pdf/1/010f019be5bb619e-0c29dfe2-cde0-4fbc-85a9-224216e52e6c-000000/m2S0Ngtua4GLJDq2eVx5NwkpN85gvEjgekDcwTyhYmw=247__;JSUlJSUlJQ!!K-Hz7m0Vt54!ii_Um56GhKhrmcj_W4aJ-LsGIGze2C0w6bYSCYyzbg0UzYwEY7L54U4Cal71Gk-PGdY4vGb1wiT6ZiU$)

 We have reviewed the requirements and reformatted/renamed files for compliance.

2. We note that the grant information you provided in the ‘Funding Information’ and ‘Financial Disclosure’ sections do not match.

When you resubmit, please ensure that you provide the correct grant numbers for the awards you received for your study in the ‘Funding Information’ section.

 We have corrected the funding information.

3. Thank you for stating the following financial disclosure:
This work was supported by project CSP #2028 from the US Department of Veterans Affairs, Cooperative Studies Program.

Please state what role the funders took in the study. If the funders had no role, please state: "The funders had no role in study design, data collection and analysis, decision to publish, or preparation of the manuscript."
If this statement is not correct you must amend it as needed.
Please include this amended Role of Funder statement in your cover letter; we will change the online submission form on your behalf.

We have updated the cover letter to state: “The funders had no role in study design, data collection and analysis, decision to publish, or preparation of the manuscript.”

4. Thank you for stating the following in the Acknowledgments Section of your manuscript:
The creation and ongoing development, management, and maintenance of the CSP#2028: Epidemiology, Immunology, and Clinical Characteristics of COVID-10 (EPIC3) study resources is supported by the Cooperative Studies Program (CSP) of the United States Department of Veterans Affairs (VA) Office of Research & Development. Most importantly, the authors gratefully acknowledge the past and continued cooperation of CSP #2028 study participants. Without their contribution, this research would not have been possible.

We note that you have provided funding information that is not currently declared in your Funding Statement. However, funding information should not appear in the Acknowledgments section or other areas of your manuscript. We will only publish funding information present in the Funding Statement section of the online submission form.
Please remove any funding-related text from the manuscript and let us know how you would like to update your Funding Statement. Currently, your Funding Statement reads as follows:
This work was supported by project CSP #2028 from the US Department of Veterans Affairs, Cooperative Studies Program.

Please include your amended statements within your cover letter; we will change the online submission form on your behalf.

We have deleted all language related to study funding from the Acknowledgments section. We confirm that the funding statement is correct as currently written.

5. Please include your full ethics statement in the ‘Methods’ section of your manuscript file. In your statement, please include the full name of the IRB or ethics committee who approved or waived your study, as well as whether or not you obtained informed written or verbal consent. If consent was waived for your study, please include this information in your statement as well.

A full ethics statement in the Methods section (page 6) reads: “The study was approved by the VA Central Institutional Review Board. Veterans or their legally authorized representative provided written informed consent for participation.”

6. When completing the data availability statement of the submission form, you indicated that you will make your data available on acceptance. We strongly recommend all authors decide on a data sharing plan before acceptance, as the process can be lengthy and hold up publication timelines. Please note that, though access restrictions are acceptable now, your entire data will need to be made freely accessible if your manuscript is accepted for publication. This policy applies to all data except where public deposition would breach compliance with the protocol approved by your research ethics board. If you are unable to adhere to our open data policy, please kindly revise your statement to explain your reasoning and we will seek the editor's input on an exemption. Please be assured that, once you have provided your new statement, the assessment of your exemption will not hold up the peer review process.

Data will not be freely accessible on acceptance. As this study was conducted within the U.S. Department of Veterans Affairs, our ability to publicly deposit the complete underlying dataset is constrained by federal regulations and VA policy governing the protection of Veteran data. Specifically, VA policy (VHA Directive 1605.01 and VA Handbook 6500) classifies research datasets containing Veteran information as sensitive and prohibits unrestricted public release, even after de-identification, without explicit VA approval. In addition, our VA Institutional Review Board-approved protocol and informed consent documents did not include provisions for public data deposition, and retroactive public release would therefore be noncompliant with both VA policy and the approved ethics protocol. Consistent with VA requirements and prevailing practices for VA-based research, we are committed to transparency and reproducibility by making the data available upon reasonable request through established VA data access mechanisms (e.g., via a VA-approved Data Use Agreement and review by the relevant VA oversight bodies). Qualified researchers may obtain access subject to VA regulatory approval, ensuring protection of Veteran privacy while enabling scientific verification.

These resources can be shared with qualified VA and non-VA investigators working for nonprofit, academic, and research centers under controlled access procedures via the study repository's application process. Data requests will be reviewed for scientific and ethical merit. If the request involves human subjects research, access is contingent upon approval by an Institutional Review Board (IRB) and, if VA research, by a VA Research and Development (R&D) Committee. Before resources are transferred, requestors may be required to sign a VA Data Use Agreement and/or Material Transfer Agreement. Requestors must be fully compliant with any applicable VA data security, privacy, confidentiality, and human subjects requirements.

7. In this instance it seems there may be acceptable restrictions in place that prevent the public sharing of your minimal data. However, in line with our goal of ensuring long-term data availability to all interested researchers, PLOS’ Data Policy states that authors cannot be the sole named individuals responsible for ensuring data access ([http://journals.plos.org/plosone/s/data-availability#loc-acceptable-data-sharing-methods](https://urldefense.com/v3/__http:/journals.plos.org/plosone/s/data-availability*loc-acceptable-data-sharing-methods__;Iw!!K-Hz7m0Vt54!ii_Um56GhKhrmcj_W4aJ-LsGIGze2C0w6bYSCYyzbg0UzYwEY7L54U4Cal71Gk-PGdY4vGb1sXGb8vE$)).

Data requests to a non-author institutional point of contact, such as a data access or ethics committee, helps guarantee long term stability and availability of data. Providing interested researchers with a durable point of contact ensures data will be accessible even if an author changes email addresses, institutions, or becomes unavailable to answer requests.

Before we proceed with your manuscript, please also provide non-author contact information (phone/email/hyperlink) for a data access committee, ethics committee, or other institutional body to which data requests may be sent. If no institutional body is available to respond to requests for your minimal data, please consider if there any institutional representatives who did not collaborate in the study, and are not listed as authors on the manuscript, who would be able to hold the data and respond to external requests for data access? If so, please provide their contact information (i.e., email address). Please also provide details on how you will ensure persistent or long-term data storage and availability.

We agree with the importance of a durable, non-author point of contact for data access requests and have designated csp2028cc@va.gov for this purpose. With respect to long-term data availability, all study data are stored on VA-approved secure servers in compliance with VA records retention, information security, and research data management policies.

8. If the reviewer comments include a recommendation to cite specific previously published works, please review and evaluate these publications to determine whether they are relevant and should be cited. There is no requirement to cite these works unless the editor has indicated otherwise.

Noted.

Additional Editor Comments:

(PLACE INSERTS IN A DIFFERENT COLOR FONT TO IDENTIFY CHANGES IN THE ARTICLE) PUT IN RED LETTERS - ANSWER THE QUESTIONS BELOW POINT BY POINT. INCLUDE THE PAGE AND LINE WHERE YOU ARE MAKING THE CHANGE. PLEASE INCLUDE ALL REQUESTS IN THE MANUSCRIPT.
Include in article

0 - Please correct grammar and spelling errors

We have done so.

1 - Compare and contrast your study with others in the most relevant world literature, particularly the recent literature.

We agree and have expanded the Discussion to better compare our findings with the most relevant recent literature, including studies identified in an updated review (March 2026). We added the following text (page 14): “Consistent with the current findings, in a 2025 retrospective analysis in a propensity score–matched cohort of U.S. active-duty service members and their families identified through the Military Health System Data Repository, SARS-CoV-2 infection was not associated with incident diabetes (HR 0.95; 99% CI: 0.75, 1.21). Whereas these two studies found no evidence of an association of SARS-CoV-2 infection with incident diabetes, large retrospective cohort studies and meta-analyses (including a very recent meta-analysis [24] have reported an elevated risk of new-onset diabetes following COVID-19 compared with non-infected controls. In those studies, associations are typically strongest among individuals with more severe disease and during the time period closest to infection. In pooled estimates from meta-analyses of retrospective studies, SARS-CoV-2 was consistently associated with a 50–80% higher risk [1-6], which differs substantially from our findings. Reasons for these conflicting results could include differences in data collection, missing data, diabetes definitions used, selection bias, surveillance bias, confounding control, or insufficient sample size. For example, because diabetes incidence peaks in the 55–64 year old age group, the older age of the SARS-CoV-2-negative participants in our study may contribute to the higher diabetes incidence in that group [25]. Likewise, the relatively low number of participants with severe SARS-CoV-2 (using hospitalization for SARS-CoV-2 as a surrogate for severity) may account for the lack of an association between SARS-CoV-2 infection and diabetes incidence because prior studies have shown that those with severe disease are at a higher risk than those with mild disease for subsequently developing diabetes.”

2 - What new information is sufficient to modify existing clinical practice?

We discuss the implications for current practice as follows (page 18): “The findings do not support changing current clinical practice, which advise routine screening beginning at age 35, and earlier or more frequent testing is recommended in those with overweight/obesity or additional risk factors, while highlighting the need for longer structured follow-up with repeated structured glycemic assessments to identify subgroups that may benefit from targeted screening or surveillance.”

3 - What are the conclusions and implications for current practice, and particularly for future research that may have a significant impact on clinical decisions?

We now consider the implications for future research (page 17) and for current practice (page 18): “To definitively determine the association of SARS-CoV-2 infection with incident diabetes, large-scale prospective studies with standardized glycemic assessment, longer follow-up, and careful phenotyping by infection severity and metabolic risk are needed to clarify the contribution of SARS-CoV-2 to diabetes risk in specific subgroups” and “The findings do not support changing current clinical practice, which advise routine screening beginning at age 35, and earlier or more frequent testing is recommended in those with overweight/obesity or additional risk factors, while highlighting the need for longer structured follow-up with repeated structured glycemic assessments to identify subgroups that may benefit from targeted screening or surveillance.”

4 - What does this study add to the literature?

We have made edits throughout the Discussion to describe the study’s contribution: evidence from a well-characterized prospective cohort with systematic follow-up, in which we did not detect an increased risk of diabetes following SARS-CoV-2 infection. We also note that modest associations cannot be excluded given the sample size and event count, helping to appropriately contextualize our findings within the existing literature.

5 – Improve - At the end of the Discussion, under the subheading "Limitations," review the limitations of your study.

At the end of the Discussion under a “Limitations” subheading, we have enhanced our presentation of the study’s limitations: “We also acknowledge several important limitations. First, although EPIC3 participants received regular study follow-up for 24 months, this follow-up did not include longitudinal fasting glucose or HbA1c testing. Most of the relevant laboratory tests were obtained as part of regular clinical care, including as part of diabetes screening or other aspects of medical care. Given that there was more random glucose testing in the SARS-CoV-2 negative group, such differences could contribute to higher diabetes ascertainment in that group. We observed similar HbA1c testing rates, which reflects active clinician-initiated diabetes screening, though differences in other testing patterns and clinical encounters could still influence detection. We did not have baseline HbA1c measurement on all participants, which limited our ability to adjust for baseline glycemia. Residual confounding may remain from factors we could not fully adjust for (e.g., BMI, baseline glycemia) as well as from imperfect measurement or categorization of covariates we included (e.g., age). While residual confounding can bias estimates in either direction, the older age distribution in the SARS-CoV-2–negative group could bias the association downward (toward a lower apparent risk among those infected), given the known positive association of age with diabetes risk. Indications for SARS-CoV-2 testing and for hospitalization were not available, raising the possibility of selection differences between -positive and -negative participants, particularly in the inpatient subgroup. However, rates of HbA1c testing were actually very similar between the groups. We had limited statistical power to detect statistically significant differences between groups; this led to large confidence intervals around our estimates which hinders interpretation of our findings. The small number of incident diabetes events limited model stability and increased the risk of overfitting. There was also inadequate power to examine the roles of vaccination or receipt of systemic corticosteroid therapy in the development of diabetes (e.g., as potential effect modifiers, or, in the case of corticosteroid therapy, as a potential mediator). Study enrollment and follow-up occurred across multiple phases of the pandemic, including periods dominated by ancestral SARS-CoV-2 strains, followed by Alpha, Delta, and Omicron variants (BA.1–BA.5), which may have distinct metabolic effects [29] that we were unable to evaluate due to sample size. Finally, because of the rigorous inclusion criteria we used to strengthen internal validity, results may not be generalizable to, e.g., Veteran populations who do not receive regular medical care and laboratory testing. Higher baseline healthcare engagement in the analytic cohort is also expected to increase rates of diabetes detection.”

6 – Improve - At the end of the limitations, under the subheading " Future directions".

We have added the following text at the end of the limitations under the subheading “Future directions”: “To definitively determine the association of SARS-CoV-2 infection with incident diabetes, large-scale prospective studies with standardized glycemic assessment, longer follow-up, and careful phenotyping by infection severity and metabolic risk are needed to clarify the contribution of SARS-CoV-2 to diabetes risk in specific subgroups.”

7 - Conclusion
Take special care to draw your conclusions only from your results and verify that your conclusions are firmly supported by your data

We have revised the Conclusion to avoid language that could be interpreted as providing definitive evidence against an association between SARS-CoV-2 infection and diabetes risk (page 18): “We found no evidence of higher risk of incident diabetes after SARS-CoV-2 infection within the observed follow-up interval, although modest associations cannot be excluded given the sample size and event count.”

8 - References
Update

We conducted an updated literature search (March 2026) and have incorporated these additional references where appropriate to reflect the current evidence base. The reference list has been updated accordingly.


Reviewers' comments:

Reviewer's Responses to Questions

**Comments to the Author**

Please use the space provided to explain your answers to the questions above. You may also include additional comments for the author, including concerns about dual publication, research ethics, or publication ethics. (Please upload your review as an attachment if it exceeds 20,000 characters)

Reviewer #1: In this paper Huang et al evalauted incidende of diabetes in a prospective cohort of patients with and without SARS-CoV-2 taking into account care-seeking patterns and other confounders.
They did not find an association between SARS-CoV-2 infection and diabetes.
This is a very neatly written paper, limitations and strenghts were very well stated and conclusions are sound.
I would only suggest that you further clarify why on your criteria for diabetes excluded treament like aGLP-1 and SGLT-2 inhibitors. I guess aGLP-1 was probably due to its use for obesity but I don't know if you might have lost some cases by eliminating these two treatments.

Thank you for this review. Within VA, criteria-for-use for GLP-1 receptor agonists and SGLT-2 inhibitors explicitly permit use for indications beyond diabetes (e.g., heart failure, chronic kidney disease, and weight management in the absence of diabetes). Because these criteria broaden prescribing beyond diabetes, they are not specific markers of incident diabetes in VA. We added the following text to the Methods (page 7): “We did not include glucose-lowering medications with common non-diabetes indications in these criteria (i.e., metformin [used for prediabetes and PCOS], GLP-1 receptor agonists [obesity], SGLT2 inhibitors [heart failure or chronic kidney disease]).”

Reviewer #2: 1. General comments
This manuscript examines whether SARS-CoV-2 infection is associated with incident diabetes in the VHA-based EPIC3 prospective longitudinal cohort and evaluates whether differences in healthcare utilization could influence diabetes detection. The study leverages RT-PCR confirmed SARS-CoV-2 testing, extensive VHA electronic health record data to exclude prevalent diabetes, and addresses potential surveillance differences by describing post-enrollment utilization patterns and adjusting for pre-enrollment laboratory testing.

Several issues limit the interpretability of the null association and should be addressed to strengthen scientific soundness.
1.1. Clarify exposure handling in the time-varying models. The manuscript treats SARS-CoV-2 status as time-varying, with participants transitioning from negative to positive during follow-up. However, several design choices (for example, the exclusion of laboratory values from day 0 to day 28, Kaplan-Meier curves stratified by baseline status, and inpatient or outpatient strata defined at enrollment) appear anchored to enrollment rather than to each infection date. This creates ambiguity about the causal contrast estimated by the Cox models and whether early post-infection hyperglycemia is consistently excluded for all infections.

We have revised and clarified the exposure handling. In updated analyses, we specify that each participant can contribute multiple SARS-CoV-2 “episodes” as follows: Participants begin in a negative or positive episode based on their initial test; subsequent positive episodes are defined by tests occurring ≥90 days after the prior episode start. Participants are not reclassified to negative after a positive episode. Inpatient/outpatient status is assigned at the start of each episode. Incident diabetes ascertainment excludes glucose values from days 0–28 of each episode. Hemoglobin A1c, diagnoses, and glucose-lowering medications are not excluded during this window. Episodes occurring after diabetes onset are not included. For analysis, each episode is considered as a follow-up period. In Cox models, SARS-CoV-2 status is time-varying, with person-time unexposed until the start of a positive episode and exposed thereafter. The 0–28 day exclusion window is applied relative to each positive episode. Kaplan–Meier curves stratified by baseline status are descriptive and do not define model risk sets.

We added the following text to the methods (page 7): “We used RT-PCR tests recorded in the CDW to define SARS-CoV-2 exposure over time. Each participant contributed one or more SARS-CoV-2 episodes defined by qualifying RT-PCR tests. Participants began in a negative episode if they had no prior positive test and ≥1 confirmed negative test within 30 days prior to enrollment, or in a positive episode if they had ≥1 positive test within the 30 days prior to enrollment. Subsequent positive episodes were defined by a positive RT-PCR test occurring ≥90 days after the start of a prior episode. SARS-CoV-2 status was treated as time-varying, with person-time classified as unexposed until the start of a positive episode and exposed thereafter.”

1.2. Low event count and model complexity. With 45 incident diabetes events overall and very few events in subgroup analyses, the multivariable Cox models (together with multiple imputation) may be vulnerable to overfitting and unstable estimates, particularly in the inpatient stratum where confidence intervals are extremely wide. The manuscript should more explicitly address this risk and consider more parsimonious modeling and or additional sensitivity analyses.

We acknowledge that, given the small number of events, estimates may be imprecise and susceptible to overfitting. However, effect estimates were similar in direction and magnitude in both minimally and fully adjusted models, suggesting that model specification did not materially influence results. Although we were cautious to limit the number of additional covariates, we also include new analyses (e.g., additionally adjusted for body mass index and education), and results were consistent with the primary analyses. We added the following text to the limitations (page 17): “The small number of incident diabetes events limited model stability and increased the risk of overfitting” and “Residual confounding may remain from factors we could not fully adjust for (e.g., BMI, baseline glycemia) as well as from imperfect measurement or categorization of covariates we included (e.g., age).”

1.3. Confounding control appears incomplete. Strong diabetes risk factors (for example, BMI, baseline glycemia such as HbA1c, and socioeconomic indicators) are available but not included in the primary adjusted models, despite clear baseline differences between exposed and unexposed groups. At minimum, the manuscript should justify the chosen adjustment set and present sensitivity analyses that include key metabolic risk factors.

Although we were cautious to limit the number of additional covariates, we now include an additional analyses adjusting for body mass index and education (as a proxy for socioeconomic status). We added the following text to the Methods (page 9) to support the use of the current adjustment set: “Given the small number of events, these adjustment sets were selected to balance parsimony with inclusion of covariates plausibly upstream of SARS-CoV-2 test positivity and diabetes risk, focusing on demographic characteristics, comorbidity burden, and prior health care utilization.”

1.4. Reporting details needed for reproducibility. Several operational definitions and analytic details require additional clarity, including fasting versus random glucose classification, construction of utilization metrics per year, censoring rules, and the variables included in the imputation model. These details are important for reproducibility and for evaluating potential biases.

Fasting glucose v. random glucose classification: Fasting glucose tests were defined as those drawn between 6:00–10:00 AM and concurrently with a low-density lipoprotein (LDL) test. The same reference list of glucose test types was used for both fasting and random measures to minimize misclassification due to inconsistent test coding.

Construction of utilization metrics: Health care utilization was assessed in the year prior to each episode start date and included number of days hospitalized, number of days with a clinical visit, and number of days with any laboratory testing. We additionally included counts of days with glucose and/or hemoglobin A1c testing to capture diabetes-specific surveillance.

Censoring rules: Follow-up for incident diabetes was censored at diagnosis of type 1 or other non–type 2 diabetes, death, withdrawal from the study, or last recorded VHA encounter. Participants contributed at most one incident diabetes event; episodes occurring after diabetes onset were classified as prevalent diabetes and not included in risk time.

Variables included in the imputation model: The multiple imputation model included demographic factors (age, sex, race, education, income), cohort and recruitment site, baseline SARS-CoV-2 status, comorbidity burden (Charlson Index), body mass index, smoking status, vaccination status, prior health care utilization (e.g., hospitalizations, visits, laboratory testing, and glucose/A1c testing in the year prior), post-episode utilization rates, and diabetes outcome indicators (event status and time-to-event).

We have added the following text to the Methods (page 6, page 8, page 9): “Fasting glucose was defined using tests drawn between 6:00 and 10:00 AM and concurrently with a low-density lipoprotein (LDL) test; all other glucose measurements were considered random”; “Follow-up was censored at diagnosis of type 1 or other non–type 2 diabetes, death, withdrawal from the study, or last recorded VHA encounter, and participants contributed at most one incident diabetes event”; and “We used the 'mice' package in R to perform multiple imputation (m = 10, maxit = 20) to address missing covariate values [18] using an imputation model including demographic characteristics, comorbidities, health care utilization measures, and diabetes outcome indicators.”

2. Introduction
The introduction provides a clear rationale. Prior evidence largely comes from retrospective EHR studies that may be influenced by differences in care-seeking and laboratory surveillance, and a prospective cohort with confirmed testing can help address these concerns. The objectives and hypotheses are stated, which is helpful.
2.1. Clarify the estimand. Please clarify whether you aim to estimate (a) diabetes risk after recent infection at enrollment, (b) diabetes risk after any infection during follow-up, or (c) diabetes risk comparing baseline test-positive and baseline test-negative participants. This distinction matters because descriptive analyses emphasize baseline stratification while the Cox models use time-varying exposure.

The primary estimand corresponds to (b) above, implemented via a time-varying exposure. We have clarified this in the Methods (specific edits above and below) and have updated Table 1 and results throughout to align descriptive summaries with this time-varying framework.

3. Methods
The methods section is generally detailed, but several items need clarification or adjustment.
3.1. Resolve an internal inconsistency in cohort counts. The analytic population is described as having 866 SARS-CoV-2 positive participants in one place, but Figure 1 and the Results and Table 1 indicate 886 positive participants at enrollment (with 344 negative). Please correct and harmonize these figures throughout.

The values for cohort counts have been updated in the text.

3.2. Clarify selection and testing context for SARS-CoV-2 negative participants. Please provide more detail on how SARS-CoV-2 negative participants were selected and why they were tested, especially in the inpatient subgroup. If test-negative inpatients were hospitalized for indications closely tied to metabolic risk (for example, cardiovascular disease), selection differences could bias comparisons even after covariate adjustment.

This is a good point. SARS-CoV-2–negative participants were identified based on SARS-CoV-2 testing history without regard to other factors; indications for testing and hospitalization are not available. We agree that differential reasons for inpatient testing could introduce selection bias. In the VA, common reasons for hospitalization include cardiovascular disease, infections, and pulmonary conditions, with cardiovascular and chronic disease–related admissions more common in older patient. During the COVID-19 period, many SARS-CoV-2–positive admissions were directly related to COVID-19 illness. However, baseline comorbidity burden, as measured by the Charlson Comorbidity Index, was similar between SARS-CoV-2–positive and –negative participants, suggesting broadly comparable underlying health status. We have added the following text to the limitations (page 16): “Indications for SARS-CoV-2 testing and hospitalization were not available, raising the possibility of selection differences between -positive and -negative participants, particularly in the inpatient subgroup.”

3.3. Specify time-varying exposure implementation. Since SARS-CoV-2 status is modeled as time-varying, please report:
3.3.1. How many baseline-negative participants later became positive and the distribution of times to conversion.

Of 386 individuals who enrolled as SARS-CoV-2 negatives, 62 converted to positive during follow-up. Of these, median conversion time was 298 days [146, 479]. We added the following text to the Results (page 10): “Among 386 participants who were SARS-CoV-2–negative at enrollment, 62 converted to positive during follow-up, with a median time to conversion of 298 days [IQR 146–479].”

3.3.2. Whether multiple infections were possible and how they were handled analytically.

Multiple SARS-CoV-2 infections were possible and were handled using the framework described above. We added the following text to the Methods (page 7): “We used RT-PCR tests recorded in the CDW to define SARS-CoV-2 exposure over time. Each participant contributed one or more SARS-CoV-2 episodes defined by qualifying RT-PCR tests.”

3.3.3. The exact time origin for follow-up in the Cox models (for example, enrollment date versus day 29 after enrollment).

The origin date of each episode was the date of the SARS-CoV-2 test that identified that episode. We added the following text to the Methods (page 7): “The time origin for each episode was the date of the SARS-CoV-2 test defining that episode.”

3.3.4. Whether delayed entry (left truncation) was used if outcomes were only counted after day 28.

Outcomes were ascertained throughout each episode; therefore, left truncation was not used. Glucose laboratory values, which may be impacted by acute illness, were excluded during the first 28 days of each episode, whereas hemoglobin A1c labs, diagnoses, and glucose-lowering prescriptions were included at any time.

3.4. Outcome definition details. Please clarify:
3.4.1. How fasting glucose was identified in EHR data, since fasting status is often not reliably encoded. If fasting status cannot be determined with confidence, consider a sensitivity analysis using criteria that do not rely on fasting classification, or analyses that rely primarily on HbA1c-based definitions.

We agree that fasting status is often not reliably captured in EHR laboratory data and did not rely on this classification. We have added the following text to the Methods (page 6): “Fasting glucose was defined using tests drawn between 6:00 and 10:00 AM and concurrently with a low-density lipoprotein (LDL) test; all other glucose measurements were considered random.”

3.4.2. The rationale for the medication list used in the diabetes definition, as it omits commonly used agents such as metformin, GLP-1 receptor agonists, and SGLT2 inhibitors. If this omission is intentional due to non-diabetes indications, please state this explicitly and consider a sensitivity analysis incorporating broader medication classes with appropriate safeguards.

Within VA, criteria-for-use for metformin, GLP-1 receptor agonists, and SGLT-2 inhibitors explicitly permit use for indications beyond diabetes (e.g., polycystic ovary syndrome, heart failure, chronic kidney disease, and weight management in the absence of diabetes). Because these criteria broaden prescribing beyond diabetes, they are not specific markers of incident diabetes in VA. We added the following text to the Methods (page 7): “We did not include glucose-lowering medications with common non-diabetes indications in these criteria (i.e., metformin [used for prediabetes and PCOS], GLP-1 receptor agonists [obesity], SGLT2 inhibitors [heart failure or chronic kidney disease]).”

3.5. Handling transient hyperglycemia. Excluding laboratory values from day 0 to day 28 after enrollment is a thoughtful approach for baseline-positive participants. However, if exposure is time-varying, the exclusion window ideally should be anchored to each infection date rather than to enrollment only. Otherwise, a baseline-negative participant who becomes infected later could have transient post-infection hyperglycemia counted as incident diabetes. Please clarify whether this could occur under your analytic approach and consider revising the strategy or adding sensitivity analyses.

We agree that the exclusion window should be anchored to each infection episode. Above, we have clarified that SARS-CoV-2 exposure was modeled as time-varying. For each positive episode, incident diabetes ascertainment excluded glucose values during days 0–28 following the episode start, thereby preventing transient post-infection hyperglycemia from being counted among participants who convert from negative to positive during follow-up. HbA1c, diagnoses, and glucose-lowering medications were included at all times.

3.6. Covariate adjustment and confounding. The adjusted models include age, sex, race, smoking, CCI, and pre-enrollment testing intensity (laboratory test days or glucose or HbA1c test days), which partially addresses confounding and surveillance. Please consider:
3.6.1. Adding BMI and baseline HbA1c (or baseline glycemic measures) to at least one adjusted model, given their strong relationship to diabetes risk and availability in your dataset.

We agree with the reviewer that BMI and baseline HbA1c may confound associations of SARS-CoV-2 status with incident diabetes. A model additionally adjusted for BMI did not materially change the estimated association (not shown). In our dataset, there was substantial missingness in baseline HbA1c; individuals with available measurements likely differ systematically from those without, raising concern for selection bias and limited interpretability of complete-case analyses; thus, we are concerned that such analyses are not representative. We added the following text to the Discussion (page 16): “Residual confounding may remain from factors we could not fully adjust for (e.g., BMI, baseline glycemia) as well as from imperfect measurement or categorization of covariates we included (e.g., age). While residual confounding can bias estimates in either direction, the older age distribution in the SARS-CoV-2–negative group could bias the association downward (toward a lower apparent risk among those infected), given the known positive association of age with diabetes risk.”

3.6.2. Providing a brief justification for the adjustment set, even if concise.

We have added the following text to the Methods (page 9): “Given the small number of events, these adjustment sets were selected to balance between parsimony with inclusion of covariates plausibly upstream of SARS-CoV-2 test positivity and diabetes risk, focusing on demographic characteristics, comorbidity burden, and prior health care utilization.”

3.6.3. Addressing site-level differences across the 16 centers, for example via robust standard errors clustered by site or stratification if feasible.

Limited numbers of sites/events precluded stratification and the use of robust standard errors. To address heterogeneity between sites, we updated the primary analyses to use a frailty model, which adds a random effect to the Cox proportional hazard models. We have updated the results and added the following text to the methods (page 8): “We fit time-varying Cox proportional hazards models with a site-level frailty term to estimate hazard ratios (HR) for incident diabetes.”

3.7. Missing data and multiple imputation. Since multiple imputation was used, please report:
3.7.1. The proportion missing for each covariate.

| **Response Table 1. Missingness of covariates by exposure and setting** | | | | | | | | | |
| --- | --- | --- | --- | --- | --- | --- | --- | --- | --- |
| **Cohort Category** | **Sex Missing** | **Race Missing** | **Education Missing** | **Rurality Missing** | **Income Missing** | **BMI Missing** | **Smoking Missing** | **CCI Missing** | **Total Episodes** |
| Negative : Inpatient | 0 (0%) | 27 (27%) | 29 (29%) | 1 (1%) | 55 (55%) | 1 (1%) | 29 (29%) | 1 (1%) | 100 |
| Negative : Outpatient | 0 (0%) | 51 (12.7%) | 40 (10%) | 9 (2.2%) | 130 (32.3%) | 12 (3%) | 39 (9.7%) | 70 (17.4%) | 402 |
| Positive : Inpatient | 0 (0%) | 50 (30.9%) | 51 (31.5%) | 25 (15.4%) | 89 (54.9%) | 0 (0%) | 50 (30.9%) | 5 (3.1%) | 162 |
| Positive : Outpatient | 3 (0.3%) | 114 (10.9%) | 97 (9.2%) | 195 (18.6%) | 371 (35.3%) | 17 (1.6%) | 94 (9%) | 240 (22.9%) | 1050 |
| **Totals** | **3 (0.2%)** | **242 (14.1%)** | **217 (12.7%)** | **230 (13.4%)** | **645 (37.6%)** | **30 (1.8%)** | **212 (12.4%)** | **316 (18.4%)** | **1714** |

We added the following text to the methods (page 9): “Overall, missingness was low for most covariates (sex 0.2%, BMI 1.8%, smoking 12.4%, race 14.1%, education 12.7%, rurality 13.4%), with some exceptions (e.g., income 37.6%).”

3.7.2. Which variables were included in the imputation model, including exposure and outcome indicators.

The imputation model included exposure (time-updated SARS-CoV-2 status) and outcome indicators (incident diabetes event status and time-to-event), along with demographic characteristics, comorbidity burden, body mass index, smoking status, vaccination status, health care utilization, and laboratory testing measures (now described in the updated Methods).

3.7.3. Any diagnostics or checks performed to support plausibility of the imputations.

We inspected trace plots to assure convergence of the imputation algorithm and density plots to compare the distributions across imputations to the observed data. There was no evidence of non-convergence, and imputed values were consistent with those observed in the complete cases.

3.8. Handling missing CCI. Coding missing CCI values as 0 could misclassify comorbidity burden. Please justify this decision or consider imputing CCI and or including a missingness indicator.

We acknowledge the concern that this strategy may reflect incomplete coding rather than true absence of comorbidity. This approach is consistent with ICD-based implementations of the Charlson index, in which the score is derived solely from recorded diagnosis codes and serves specifically to capture documented comorbid conditions. ICD-based Charlson Comorbidity Index algorithms demonstrate good predictive validity across coding systems, supporting their use in administrative data despite reliance on recorded diagnoses (PMID: 21619668). In our dataset, CCI was missing in 18.4% of participants at baseline. For analysis, if CCI was missing at baseline, the value was imputed. We added the following text to the Methods: (page 8) “The Charlson Comorbidity Index (CCI), a weighted summary measure of 17 chronic conditions used to quantify overall documented comorbidity burden.”

3.9. Follow-up and censoring. Please define precisely how follow-up ended (incident diabetes, death, last VA encounter, end of data availability, withdrawal, etc.).

Follow-up ended at the earliest of the following: diagnosis of type 1 or other non–type 2 diabetes, death, withdrawal from the study, or last recorded VHA encounter. We added the following text to Methods (page 8): “Follow-up was censored at diagnosis of type 1 or other non–type 2 diabetes, death, withdrawal from the study, or last recorded VHA encounter, and participants contributed at most one incident diabetes event.”

3.10. Competing risks. Given the population and follow-up duration, consider whether death is a competing risk that could meaningfully influence results. Even if not formally modeled, reporting mortality or censoring patterns would help readers judge potential bias.

We fit Fine-Gray models (not shown) with death as a competing risk. While subdistribution hazard ratios for SARS-CoV-2 status were lower, they were still not significantly below 1 and results were overall consistent with the Cox proportional hazard models.


4. Results
The results are clearly organized, and the descriptive comparison of utilization patterns is helpful. A few additions would strengthen interpretability.
4.1. Report absolute rates and risk differences. In addition to cumulative proportions with incident diabetes (3.4% vs 4.4%), please report incidence rates (for example, per 1,000 person-years) and an absolute risk difference over a clinically meaningful time horizon. This will help readers understand the magnitude of any potential association even when estimates are imprecise.

We appreciate this suggestion. We now report incidence rates and counts of incident diabetes.

4.2. Clarify summary statistics in utilization tables. Table 1 reports medians with interquartile ranges, but Table 2 provides single utilization values per 365 days without stating whether these are means, medians, or model-based estimates. Please specify the statistic and consider adding dispersion (IQR or SD), since utilization measures are typically skewed.

We have revised Table 2 as recommended.

4.3. Report missingness in key covariates. Please report the number of participants with missing BMI, smoking status, income, and other covariates, and whether imputation materially changed the analytic sample or key estimates.

Please see the response to 3.7.3 above. We inspected density plots to compare the distributions across imputations to the observed data. Imputed values were consistent with those observed in the complete cases.

4.4. Time-varying exposure transparency. If exposure is time-varying, readers need to know how often and when exposure changed. Reporting how many baseline-negative participants became infected during follow-up, and how they contributed person-time, is essential.

Of 386 individuals who enrolled as SARS-CoV-2 negatives, 62 converted to positive during follow-up. Of these, median conversion time was 298 days [146, 479]. We added the following text to the Results (page 10): “Among 386 participants who were SARS-CoV-2–negative at enrollment, 62 converted to positive during follow-up, with a median time to conversion of 298 days [IQR 146–479].”

4.5. De-emphasize post hoc power. Post hoc power calculations can be misleading because confidence intervals already convey uncertainty. Consider replacing this with a discussion of detectable effect sizes given the observed event counts, or interpreting the confidence intervals directly.

We agree with this comment and have removed the post-hoc power calculations.

5. Discussion
The discussion is balanced and acknowledges important limitations, including observational design, lack of protocolized longitudinal glycemic testing, limited power, and inability to assess effect modification by vaccination, steroids, or variant. The emphasis on surveillance bias is appropriate.
5.1. Align interpretation with uncertainty. While you note that you did not replicate prior reports of increased risk, the adjusted confidence intervals (for example, 0.31 to 1.56 overall) still allow for a clinically meaningful increase in risk. Consider wording that emphasizes “no evidence of increased risk in this cohort” rather than implying evidence of no association.

We have revised the Conclusion to avoid language that could be interpreted as providing definitive evidence against an association between SARS-CoV-2 infection and diabetes risk (page 18): “We found no evidence of higher risk of incident diabetes after SARS-CoV-2 infection within the observed follow-up interval, although modest associations cannot be excluded given the sample size and event count.”

5.2. Discuss residual confounding more explicitly. Baseline differences (age, smoking, vaccination, income) and the absence of adjustment for BMI and baseline glycemia should be discussed as potential sources of residual confounding that could bias estimates in either direction.

We now include explicit discussion of residual confounding (page 16): “Residual confounding may remain from factors we could not fully adjust for (e.g., baseline glycemia) as well as from imperfect measurement or categorization of covariates we included (e.g., age). While residual confounding can bias estimates in either direction, the older age distribution in the SARS-CoV-2–negative group could bias the association downward (toward a lower apparent risk among those infected), given the known positive association of age with diabetes risk.”

5.3. Clarify the link between utilization patterns and ascertainment. You report lower overall utilization but similar HbA1c testing rates in the SARS-CoV-2 positive group. It would strengthen the discussion to address whether HbA1c testing is the most relevant surveillance metric for diabetes detection in this context and whether differences in other testing patterns (for example, glucose testing frequency or clinical encounters) could still influence ascertainment.

We added the following text to the Discussion (page 16): “We observed similar HbA1c testing rates, which reflects active clinician-initiated diabetes screening, though differences in other testing patterns and clinical encounters could still influence detection.”

5.4. Selection implications of baseline lab requirements. Requiring a recent non-diabetic laboratory test likely increases internal validity but may select for Veterans who are more engaged with care. A brief discussion of how this could influence both utilization and diabetes detection would be helpful.

We added the following text to the Discussion (page 16): “Requiring a recent laboratory test that excluded prevalent diabetes increased internal validity but may have selected for individuals more engaged in care.”

6. Conclusion
The conclusion appropriately notes similar HbA1c testing rates and limited power.
6.1. Please avoid statements that could be read as definitive evidence that SARS-CoV-2 is not associated with diabetes risk. Instead, emphasize that this study did not detect an increased risk, while acknowledging that modest associations cannot be excluded given the sample size and event count.

We have revised the Conclusion to avoid language that could be interpreted as providing definitive evidence against an association between SARS-CoV-2 infection and diabetes risk (page 18): “We found no evidence of higher risk of incident diabetes after SARS-CoV-2 infection within the observed follow-up interval, although modest associations cannot be excluded given the sample size and event count.”

Thank you for the opportunity to review this manuscript. I hope these comments are helpful in strengthening the clarity, reproducibility, and interpretation of the study.
