# Supplementary material for: Association of SARS-CoV-2 infection with incident diabetes among U.S. Veterans in a prospective longitudinal cohort
Source: PLoS One. 2026 Jun 26;21(6):e0351992. doi: 10.1371/journal.pone.0351992 (PMC13308785; doi:10.1371/journal.pone.0351992)
Supplement: Attachment — Submitted filename: response to reviewers_062826.docx [file pone.0351992.s007.docx]

28 May 2026

Dear editors:

Thank you for your thoughtful review and for the opportunity to revise this manuscript. We respond to the reviewers’ comments point-by-point below.

Great gratitude,

Luke

Pandora Lucrezia “Luke” Wander, MD, MS, FACP

Associate Professor of Medicine

Adjunct Associate Professor of Epidemiology & Public Health Genetics

University of Washington

Staff Physician

Veterans Affairs Puget Sound Health Care System

Journal Requirements:

Reviewers' comments:

6. Review Comments to the Author

Please use the space provided to explain your answers to the questions above. You may also include additional comments for the author, including concerns about dual publication, research ethics, or publication ethics. (Please upload your review as an attachment if it exceeds 20,000 characters)

Reviewer #2: Thank you for the careful revision and detailed point-by-point response. The revised manuscript is substantially improved, and most of my prior concerns have been addressed satisfactorily. In particular, the authors have clarified the time-varying exposure framework, reported the number and timing of participants who converted from SARS-CoV-2 negative to positive during follow-up, added incidence rates, expanded the description of missing data and imputation, included an additional model adjusted for BMI and education, accounted for site-level differences using a frailty term, and appropriately tempered the interpretation of the null findings.

I have no remaining major concerns regarding the scientific soundness of the work. However, I recommend that a few minor issues be corrected before acceptance:

1. The conclusion appears to retain an outdated statement that incident diabetes occurred in 3.7% of participants over an average of roughly 1,000 days of follow-up. This is inconsistent with the revised Results and Table 1, which report incident diabetes in 6.5% of SARS-CoV-2 positive and 6.7% of SARS-CoV-2 negative participants, with median follow-up of approximately 4 years and corresponding incidence rates of 16.1 and 21.2 cases per 1,000 person-years. Please harmonize the conclusion with the revised results.

Thanks for catching this. We have updated the conclusion to state the following (page 16): “Incident diabetes was relatively common, with rates of 16.1 and 21.2 cases per 1,000 person-years among SARS-CoV-2 positive and negative participants, respectively.”

2. The description of Figure 2 and the Kaplan-Meier analysis remains somewhat ambiguous. The response states that the Kaplan-Meier curves are descriptive and stratified by baseline SARS-CoV-2 status, whereas the manuscript refers to “extended Kaplan-Meier” curves and also emphasizes time-varying SARS-CoV-2 exposure. Please clarify whether Figure 2 represents baseline-stratified descriptive curves or a time-varying survival display, and ensure that the figure caption, Methods, and Results are aligned.

Apologies for the continued confusion. SARS-CoV-2 status was modified as a time-varying exposure. We have revised the methods (page 8) to read: “We used extended Kaplan-Meier analysis to estimate diabetes-free survival stratified by time-varying SARS-CoV-2 status.” We have revised the Fig. 2 legend to read: “Overall and stratified by recruitment setting and time-varying SARS-CoV-2 status.”

3. Please correct remaining minor typographical and formatting errors. For example, Table 1 reports “103 (125%)” for one education category, which appears to be a missing decimal point. The conclusion also contains minor grammatical issues, such as “current clinical practice, which advise.”

We have corrected the typographical and formatting errors identified.

After these minor corrections, I believe the manuscript will be suitable for publication.

Kind regards.
