# Supplementary material for: Association of SARS-CoV-2 infection with incident diabetes among U.S. Veterans in a prospective longitudinal cohort
Source: PLoS One. 2026 Jun 26;21(6):e0351992. doi: 10.1371/journal.pone.0351992 (PMC13308785; doi:10.1371/journal.pone.0351992)
Supplement: Peer review history

# Peer review: Decision Letter 0

**DOI:** 10.1371/journal.pone.0351992.r001

**Authors:** Leonardo Roever

22 Jan 2026

PONE-D-25-67341

Association of SARS-CoV-2 infection with incident diabetes among U.S. Veterans in a prospective longitudinal cohort

PLOS One

Dear Dr. Wander,

Thank you for submitting your manuscript to PLOS ONE. After careful consideration, we feel that it has merit but does not fully meet PLOS ONE’s publication criteria as it currently stands. Therefore, we invite you to submit a revised version of the manuscript that addresses the points raised during the review process.

Please submit your revised manuscript by Mar 06 2026 11:59PM. If you will need more time than this to complete your revisions, please reply to this message or contact the journal office at plosone@plos.org. When you're ready to submit your revision, log on to https://www.editorialmanager.com/pone/ and select the 'Submissions Needing Revision' folder to locate your manuscript file.

Please include the following items when submitting your revised manuscript:

- A letter that responds to each point raised by the academic editor and reviewer(s). You should upload this letter as a separate file labeled 'Response to Reviewers'.
- A marked-up copy of your manuscript that highlights changes made to the original version. You should upload this as a separate file labeled 'Revised Manuscript with Track Changes'.
- An unmarked version of your revised paper without tracked changes. You should upload this as a separate file labeled 'Manuscript'.

If you would like to make changes to your financial disclosure, please include your updated statement in your cover letter. Guidelines for resubmitting your figure files are available below the reviewer comments at the end of this letter.

If applicable, we recommend that you deposit your laboratory protocols in protocols.io to enhance the reproducibility of your results. Protocols.io assigns your protocol its own identifier (DOI) so that it can be cited independently in the future. For instructions see: https://journals.plos.org/plosone/s/submission-guidelines#loc-laboratory-protocols. Additionally, PLOS ONE offers an option for publishing peer-reviewed Lab Protocol articles, which describe protocols hosted on protocols.io. Read more information on sharing protocols at https://plos.org/protocols?utm_medium=editorial-email&utm_source=authorletters&utm_campaign=protocols.

We look forward to receiving your revised manuscript.

Kind regards,

Leonardo Roever PhD, MBA

Academic Editor

PLOS One

Journal Requirements:

When submitting your revision, we need you to address these additional requirements.

1. Please ensure that your manuscript meets PLOS ONE's style requirements, including those for file naming. The PLOS ONE style templates can be found at

https://journals.plos.org/plosone/s/file?id=wjVg/PLOSOne_formatting_sample_main_body.pdf and

https://journals.plos.org/plosone/s/file?id=ba62/PLOSOne_formatting_sample_title_authors_affiliations.pdf

2. We note that the grant information you provided in the ‘Funding Information’ and ‘Financial Disclosure’ sections do not match.

When you resubmit, please ensure that you provide the correct grant numbers for the awards you received for your study in the ‘Funding Information’ section.

3. Thank you for stating the following financial disclosure:

This work was supported by project CSP #2028 from the US Department of Veterans Affairs, Cooperative Studies Program.

Please state what role the funders took in the study. If the funders had no role, please state: "The funders had no role in study design, data collection and analysis, decision to publish, or preparation of the manuscript."

If this statement is not correct you must amend it as needed.

Please include this amended Role of Funder statement in your cover letter; we will change the online submission form on your behalf.

4. Thank you for stating the following in the Acknowledgments Section of your manuscript:

The creation and ongoing development, management, and maintenance of the CSP#2028: Epidemiology, Immunology, and Clinical Characteristics of COVID-10 (EPIC3) study resources is supported by the Cooperative Studies Program (CSP) of the United States Department of Veterans Affairs (VA) Office of Research & Development. Most importantly, the authors gratefully acknowledge the past and continued cooperation of CSP #2028 study participants. Without their contribution, this research would not have been possible.

We note that you have provided funding information that is not currently declared in your Funding Statement. However, funding information should not appear in the Acknowledgments section or other areas of your manuscript. We will only publish funding information present in the Funding Statement section of the online submission form.

Please remove any funding-related text from the manuscript and let us know how you would like to update your Funding Statement. Currently, your Funding Statement reads as follows:

This work was supported by project CSP #2028 from the US Department of Veterans Affairs, Cooperative Studies Program.

Please include your amended statements within your cover letter; we will change the online submission form on your behalf.

5. Please include your full ethics statement in the ‘Methods’ section of your manuscript file. In your statement, please include the full name of the IRB or ethics committee who approved or waived your study, as well as whether or not you obtained informed written or verbal consent. If consent was waived for your study, please include this information in your statement as well.

6. When completing the data availability statement of the submission form, you indicated that you will make your data available on acceptance. We strongly recommend all authors decide on a data sharing plan before acceptance, as the process can be lengthy and hold up publication timelines. Please note that, though access restrictions are acceptable now, your entire data will need to be made freely accessible if your manuscript is accepted for publication. This policy applies to all data except where public deposition would breach compliance with the protocol approved by your research ethics board. If you are unable to adhere to our open data policy, please kindly revise your statement to explain your reasoning and we will seek the editor's input on an exemption. Please be assured that, once you have provided your new statement, the assessment of your exemption will not hold up the peer review process.

7. In this instance it seems there may be acceptable restrictions in place that prevent the public sharing of your minimal data. However, in line with our goal of ensuring long-term data availability to all interested researchers, PLOS’ Data Policy states that authors cannot be the sole named individuals responsible for ensuring data access (http://journals.plos.org/plosone/s/data-availability#loc-acceptable-data-sharing-methods).

Data requests to a non-author institutional point of contact, such as a data access or ethics committee, helps guarantee long term stability and availability of data. Providing interested researchers with a durable point of contact ensures data will be accessible even if an author changes email addresses, institutions, or becomes unavailable to answer requests.

Before we proceed with your manuscript, please also provide non-author contact information (phone/email/hyperlink) for a data access committee, ethics committee, or other institutional body to which data requests may be sent. If no institutional body is available to respond to requests for your minimal data, please consider if there any institutional representatives who did not collaborate in the study, and are not listed as authors on the manuscript, who would be able to hold the data and respond to external requests for data access? If so, please provide their contact information (i.e., email address). Please also provide details on how you will ensure persistent or long-term data storage and availability.

8. If the reviewer comments include a recommendation to cite specific previously published works, please review and evaluate these publications to determine whether they are relevant and should be cited. There is no requirement to cite these works unless the editor has indicated otherwise.

Additional Editor Comments:

(PLACE INSERTS IN A DIFFERENT COLOR FONT TO IDENTIFY CHANGES IN THE ARTICLE)

PUT IN RED LETTERS - ANSWER THE QUESTIONS BELOW POINT BY POINT.

INCLUDE THE PAGE AND LINE WHERE YOU ARE MAKING THE CHANGE.

PLEASE INCLUDE ALL REQUESTS IN THE MANUSCRIPT.

Include in article

0 - Please correct grammar and spelling errors

1 - Compare and contrast your study with others in the most relevant world literature, particularly the recent literature.

2 - What new information is sufficient to modify existing clinical practice?

3 - What are the conclusions and implications for current practice, and particularly for future research that may have a significant impact on clinical decisions?

4 - What does this study add to the literature?

5 – Improve - At the end of the Discussion, under the subheading "Limitations," review the limitations of your study.

6 – Improve - At the end of the limitations, under the subheading " Future directions".

7 - Conclusion

Take special care to draw your conclusions only from your results and verify that your conclusions are firmly supported by your data

8 - References

Update

[Note: HTML markup is below. Please do not edit.]

Reviewers' comments:

Reviewer's Responses to Questions

**Comments to the Author**

1. Is the manuscript technically sound, and do the data support the conclusions?

The manuscript must describe a technically sound piece of scientific research with data that supports the conclusions. Experiments must have been conducted rigorously, with appropriate controls, replication, and sample sizes. The conclusions must be drawn appropriately based on the data presented.

Reviewer #1: Yes

Reviewer #2: Yes

**********

2. Has the statistical analysis been performed appropriately and rigorously?

Reviewer #1: Yes

Reviewer #2: Yes

**********

3. Have the authors made all data underlying the findings in their manuscript fully available?

The PLOS Data policy requires authors to make all data underlying the findings described in their manuscript fully available without restriction, with rare exception (please refer to the Data Availability Statement in the manuscript PDF file). The data should be provided as part of the manuscript or its supporting information, or deposited to a public repository. For example, in addition to summary statistics, the data points behind means, medians and variance measures should be available. If there are restrictions on publicly sharing data—e.g. participant privacy or use of data from a third party—those must be specified.

Reviewer #1: Yes

Reviewer #2: No

**********

4. Is the manuscript presented in an intelligible fashion and written in standard English?

PLOS ONE does not copyedit accepted manuscripts, so the language in submitted articles must be clear, correct, and unambiguous. Any typographical or grammatical errors should be corrected at revision, so please note any specific errors here.

Reviewer #1: Yes

Reviewer #2: Yes

**********

5. Review Comments to the Author

Please use the space provided to explain your answers to the questions above. You may also include additional comments for the author, including concerns about dual publication, research ethics, or publication ethics. (Please upload your review as an attachment if it exceeds 20,000 characters)

Reviewer #1: In this paper Huang et al evalauted incidende of diabetes in a prospective cohort of patients with and without SARS-CoV-2 taking into account care-seeking patterns and other confounders.

They did not find an association between SARS-CoV-2 infection and diabetes.

This is a very neatly written paper, limitations and strenghts were very well stated and conclusions are sound.

I would only suggest that you further clarify why on your criteria for diabetes excluded treament like aGLP-1 and SGLT-2 inhibitors. I guess aGLP-1 was probably due to its use for obesity but I don't know if you might have lost some cases by eliminating these two treatments.

Reviewer #2: 1. General comments

This manuscript examines whether SARS-CoV-2 infection is associated with incident diabetes in the VHA-based EPIC3 prospective longitudinal cohort and evaluates whether differences in healthcare utilization could influence diabetes detection. The study leverages RT-PCR confirmed SARS-CoV-2 testing, extensive VHA electronic health record data to exclude prevalent diabetes, and addresses potential surveillance differences by describing post-enrollment utilization patterns and adjusting for pre-enrollment laboratory testing.

Several issues limit the interpretability of the null association and should be addressed to strengthen scientific soundness.

1.1. Clarify exposure handling in the time-varying models. The manuscript treats SARS-CoV-2 status as time-varying, with participants transitioning from negative to positive during follow-up. However, several design choices (for example, the exclusion of laboratory values from day 0 to day 28, Kaplan-Meier curves stratified by baseline status, and inpatient or outpatient strata defined at enrollment) appear anchored to enrollment rather than to each infection date. This creates ambiguity about the causal contrast estimated by the Cox models and whether early post-infection hyperglycemia is consistently excluded for all infections.

1.2. Low event count and model complexity. With 45 incident diabetes events overall and very few events in subgroup analyses, the multivariable Cox models (together with multiple imputation) may be vulnerable to overfitting and unstable estimates, particularly in the inpatient stratum where confidence intervals are extremely wide. The manuscript should more explicitly address this risk and consider more parsimonious modeling and or additional sensitivity analyses.

1.3. Confounding control appears incomplete. Strong diabetes risk factors (for example, BMI, baseline glycemia such as HbA1c, and socioeconomic indicators) are available but not included in the primary adjusted models, despite clear baseline differences between exposed and unexposed groups. At minimum, the manuscript should justify the chosen adjustment set and present sensitivity analyses that include key metabolic risk factors.

1.4. Reporting details needed for reproducibility. Several operational definitions and analytic details require additional clarity, including fasting versus random glucose classification, construction of utilization metrics per year, censoring rules, and the variables included in the imputation model. These details are important for reproducibility and for evaluating potential biases.

2. Introduction

The introduction provides a clear rationale. Prior evidence largely comes from retrospective EHR studies that may be influenced by differences in care-seeking and laboratory surveillance, and a prospective cohort with confirmed testing can help address these concerns. The objectives and hypotheses are stated, which is helpful.

2.1. Clarify the estimand. Please clarify whether you aim to estimate (a) diabetes risk after recent infection at enrollment, (b) diabetes risk after any infection during follow-up, or (c) diabetes risk comparing baseline test-positive and baseline test-negative participants. This distinction matters because descriptive analyses emphasize baseline stratification while the Cox models use time-varying exposure.

3. Methods

The methods section is generally detailed, but several items need clarification or adjustment.

3.1. Resolve an internal inconsistency in cohort counts. The analytic population is described as having 866 SARS-CoV-2 positive participants in one place, but Figure 1 and the Results and Table 1 indicate 886 positive participants at enrollment (with 344 negative). Please correct and harmonize these figures throughout.

3.2. Clarify selection and testing context for SARS-CoV-2 negative participants. Please provide more detail on how SARS-CoV-2 negative participants were selected and why they were tested, especially in the inpatient subgroup. If test-negative inpatients were hospitalized for indications closely tied to metabolic risk (for example, cardiovascular disease), selection differences could bias comparisons even after covariate adjustment.

3.3. Specify time-varying exposure implementation. Since SARS-CoV-2 status is modeled as time-varying, please report:

3.3.1. How many baseline-negative participants later became positive and the distribution of times to conversion.

3.3.2. Whether multiple infections were possible and how they were handled analytically.

3.3.3. The exact time origin for follow-up in the Cox models (for example, enrollment date versus day 29 after enrollment).

3.3.4. Whether delayed entry (left truncation) was used if outcomes were only counted after day 28.

3.4. Outcome definition details. Please clarify:

3.4.1. How fasting glucose was identified in EHR data, since fasting status is often not reliably encoded. If fasting status cannot be determined with confidence, consider a sensitivity analysis using criteria that do not rely on fasting classification, or analyses that rely primarily on HbA1c-based definitions.

3.4.2. The rationale for the medication list used in the diabetes definition, as it omits commonly used agents such as metformin, GLP-1 receptor agonists, and SGLT2 inhibitors. If this omission is intentional due to non-diabetes indications, please state this explicitly and consider a sensitivity analysis incorporating broader medication classes with appropriate safeguards.

3.5. Handling transient hyperglycemia. Excluding laboratory values from day 0 to day 28 after enrollment is a thoughtful approach for baseline-positive participants. However, if exposure is time-varying, the exclusion window ideally should be anchored to each infection date rather than to enrollment only. Otherwise, a baseline-negative participant who becomes infected later could have transient post-infection hyperglycemia counted as incident diabetes. Please clarify whether this could occur under your analytic approach and consider revising the strategy or adding sensitivity analyses.

3.6. Covariate adjustment and confounding. The adjusted models include age, sex, race, smoking, CCI, and pre-enrollment testing intensity (laboratory test days or glucose or HbA1c test days), which partially addresses confounding and surveillance. Please consider:

3.6.1. Adding BMI and baseline HbA1c (or baseline glycemic measures) to at least one adjusted model, given their strong relationship to diabetes risk and availability in your dataset.

3.6.2. Providing a brief justification for the adjustment set, even if concise.

3.6.3. Addressing site-level differences across the 16 centers, for example via robust standard errors clustered by site or stratification if feasible.

3.7. Missing data and multiple imputation. Since multiple imputation was used, please report:

3.7.1. The proportion missing for each covariate.

3.7.2. Which variables were included in the imputation model, including exposure and outcome indicators.

3.7.3. Any diagnostics or checks performed to support plausibility of the imputations.

3.8. Handling missing CCI. Coding missing CCI values as 0 could misclassify comorbidity burden. Please justify this decision or consider imputing CCI and or including a missingness indicator.

3.9. Follow-up and censoring. Please define precisely how follow-up ended (incident diabetes, death, last VA encounter, end of data availability, withdrawal, etc.).

3.10. Competing risks. Given the population and follow-up duration, consider whether death is a competing risk that could meaningfully influence results. Even if not formally modeled, reporting mortality or censoring patterns would help readers judge potential bias.

4. Results

The results are clearly organized, and the descriptive comparison of utilization patterns is helpful. A few additions would strengthen interpretability.

4.1. Report absolute rates and risk differences. In addition to cumulative proportions with incident diabetes (3.4% vs 4.4%), please report incidence rates (for example, per 1,000 person-years) and an absolute risk difference over a clinically meaningful time horizon. This will help readers understand the magnitude of any potential association even when estimates are imprecise.

4.2. Clarify summary statistics in utilization tables. Table 1 reports medians with interquartile ranges, but Table 2 provides single utilization values per 365 days without stating whether these are means, medians, or model-based estimates. Please specify the statistic and consider adding dispersion (IQR or SD), since utilization measures are typically skewed.

4.3. Report missingness in key covariates. Please report the number of participants with missing BMI, smoking status, income, and other covariates, and whether imputation materially changed the analytic sample or key estimates.

4.4. Time-varying exposure transparency. If exposure is time-varying, readers need to know how often and when exposure changed. Reporting how many baseline-negative participants became infected during follow-up, and how they contributed person-time, is essential.

4.5. De-emphasize post hoc power. Post hoc power calculations can be misleading because confidence intervals already convey uncertainty. Consider replacing this with a discussion of detectable effect sizes given the observed event counts, or interpreting the confidence intervals directly.

5. Discussion

The discussion is balanced and acknowledges important limitations, including observational design, lack of protocolized longitudinal glycemic testing, limited power, and inability to assess effect modification by vaccination, steroids, or variant. The emphasis on surveillance bias is appropriate.

5.1. Align interpretation with uncertainty. While you note that you did not replicate prior reports of increased risk, the adjusted confidence intervals (for example, 0.31 to 1.56 overall) still allow for a clinically meaningful increase in risk. Consider wording that emphasizes “no evidence of increased risk in this cohort” rather than implying evidence of no association.

5.2. Discuss residual confounding more explicitly. Baseline differences (age, smoking, vaccination, income) and the absence of adjustment for BMI and baseline glycemia should be discussed as potential sources of residual confounding that could bias estimates in either direction.

5.3. Clarify the link between utilization patterns and ascertainment. You report lower overall utilization but similar HbA1c testing rates in the SARS-CoV-2 positive group. It would strengthen the discussion to address whether HbA1c testing is the most relevant surveillance metric for diabetes detection in this context and whether differences in other testing patterns (for example, glucose testing frequency or clinical encounters) could still influence ascertainment.

5.4. Selection implications of baseline lab requirements. Requiring a recent non-diabetic laboratory test likely increases internal validity but may select for Veterans who are more engaged with care. A brief discussion of how this could influence both utilization and diabetes detection would be helpful.

6. Conclusion

The conclusion appropriately notes similar HbA1c testing rates and limited power.

6.1. Please avoid statements that could be read as definitive evidence that SARS-CoV-2 is not associated with diabetes risk. Instead, emphasize that this study did not detect an increased risk, while acknowledging that modest associations cannot be excluded given the sample size and event count.

Thank you for the opportunity to review this manuscript. I hope these comments are helpful in strengthening the clarity, reproducibility, and interpretation of the study.

**********

6. PLOS authors have the option to publish the peer review history of their article (what does this mean?). If published, this will include your full peer review and any attached files.

If you choose “no”, your identity will remain anonymous but your review may still be made public.

**Do you want your identity to be public for this peer review?**  For information about this choice, including consent withdrawal, please see our Privacy Policy.

Reviewer #1: **Yes:**  Mercedes Aguilar-Soto

Reviewer #2: No

**********

[NOTE: If reviewer comments were submitted as an attachment file, they will be attached to this email and accessible via the submission site. Please log into your account, locate the manuscript record, and check for the action link "View Attachments". If this link does not appear, there are no attachment files.]

To ensure your figures meet our technical requirements, please review our figure guidelines: https://journals.plos.org/plosone/s/figures

You may also use PLOS’s free figure tool, NAAS, to help you prepare publication quality figures: https://journals.plos.org/plosone/s/figures#loc-tools-for-figure-preparation.

NAAS will assess whether your figures meet our technical requirements by comparing each figure against our figure specifications.

# Author comment: Author response to Decision Letter 1

**DOI:** 10.1371/journal.pone.0351992.r002

7 May 2026

4 May 2026

Dear editors:

Thank you for your thoughtful review and for the opportunity to revise this manuscript. We respond to the reviewers’ comments point-by-point in an attachment titled response to reviewers.

Great gratitude,

Luke

Attachment Submitted filename: response to reviewers_050426.docx

# Peer review: Decision Letter 1

**DOI:** 10.1371/journal.pone.0351992.r003

**Authors:** Leonardo Roever, Felix Bongomin

26 May 2026

PONE-D-25-67341R1

Association of SARS-CoV-2 infection with incident diabetes among U.S. Veterans in a prospective longitudinal cohort

PLOS One

Dear Dr. Wander,

Thank you for submitting your manuscript to PLOS ONE. After careful consideration, we feel that it has merit but does not fully meet PLOS ONE’s publication criteria as it currently stands. Therefore, we invite you to submit a revised version of the manuscript that addresses the points raised during the review process.

Please submit your revised manuscript by Jul 10 2026 11:59PM. If you will need more time than this to complete your revisions, please reply to this message or contact the journal office at plosone@plos.org. When you're ready to submit your revision, log on to https://www.editorialmanager.com/pone/ and select the 'Submissions Needing Revision' folder to locate your manuscript file.

Please include the following items when submitting your revised manuscript:

- A letter that responds to each point raised by the academic editor and reviewer(s). You should upload this letter as a separate file labeled 'Response to Reviewers'.
- A marked-up copy of your manuscript that highlights changes made to the original version. You should upload this as a separate file labeled 'Revised Manuscript with Track Changes'.
- An unmarked version of your revised paper without tracked changes. You should upload this as a separate file labeled 'Manuscript'.

If you would like to make changes to your financial disclosure, please include your updated statement in your cover letter. Guidelines for resubmitting your figure files are available below the reviewer comments at the end of this letter.

If applicable, we recommend that you deposit your laboratory protocols in protocols.io to enhance the reproducibility of your results. Protocols.io assigns your protocol its own identifier (DOI) so that it can be cited independently in the future. For instructions see: https://journals.plos.org/plosone/s/submission-guidelines#loc-laboratory-protocols. Additionally, PLOS ONE offers an option for publishing peer-reviewed Lab Protocol articles, which describe protocols hosted on protocols.io. Read more information on sharing protocols at https://plos.org/protocols?utm_medium=editorial-email&utm_source=authorletters&utm_campaign=protocols.

As the corresponding author, your ORCID iD is verified in the submission system and will appear in the published article. PLOS supports the use of ORCID, and we encourage all coauthors to register for an ORCID iD and use it as well. Please encourage your coauthors to verify their ORCID iD within the submission system before final acceptance, as unverified ORCID iDs will not appear in the published article. *Only*  the individual author can complete the verification step; PLOS staff *cannot*  verify ORCID iDs on behalf of authors.

We look forward to receiving your revised manuscript.

Kind regards,

Felix Bongomin, MB ChB, Ph.D., FECMM

Academic Editor

PLOS One

Journal Requirements:

1. If the reviewer comments include a recommendation to cite specific previously published works, please review and evaluate these publications to determine whether they are relevant and should be cited. There is no requirement to cite these works unless the editor has indicated otherwise.

2. Please review your reference list to ensure that it is complete and correct. If you have cited papers that have been retracted, please include the rationale for doing so in the manuscript text, or remove these references and replace them with relevant current references. Any changes to the reference list should be mentioned in the rebuttal letter that accompanies your revised manuscript. If you need to cite a retracted article, indicate the article’s retracted status in the References list and also include a citation and full reference for the retraction notice.

[Note: HTML markup is below. Please do not edit.]

Reviewers' comments:

Reviewer's Responses to Questions

**Comments to the Author**

1. If the authors have adequately addressed your comments raised in a previous round of review and you feel that this manuscript is now acceptable for publication, you may indicate that here to bypass the “Comments to the Author” section, enter your conflict of interest statement in the “Confidential to Editor” section, and submit your "Accept" recommendation.

Reviewer #2: (No Response)

**********

2. Is the manuscript technically sound, and do the data support the conclusions?

The manuscript must describe a technically sound piece of scientific research with data that supports the conclusions. Experiments must have been conducted rigorously, with appropriate controls, replication, and sample sizes. The conclusions must be drawn appropriately based on the data presented.

Reviewer #2: Yes

**********

3. Has the statistical analysis been performed appropriately and rigorously?

Reviewer #2: Yes

**********

4. Have the authors made all data underlying the findings in their manuscript fully available?

The PLOS Data policy requires authors to make all data underlying the findings described in their manuscript fully available without restriction, with rare exception (please refer to the Data Availability Statement in the manuscript PDF file). The data should be provided as part of the manuscript or its supporting information, or deposited to a public repository. For example, in addition to summary statistics, the data points behind means, medians and variance measures should be available. If there are restrictions on publicly sharing data—e.g. participant privacy or use of data from a third party—those must be specified.

Reviewer #2: No

**********

5. Is the manuscript presented in an intelligible fashion and written in standard English?

PLOS ONE does not copyedit accepted manuscripts, so the language in submitted articles must be clear, correct, and unambiguous. Any typographical or grammatical errors should be corrected at revision, so please note any specific errors here.

Reviewer #2: Yes

**********

6. Review Comments to the Author

Please use the space provided to explain your answers to the questions above. You may also include additional comments for the author, including concerns about dual publication, research ethics, or publication ethics. (Please upload your review as an attachment if it exceeds 20,000 characters)

Reviewer #2: Thank you for the careful revision and detailed point-by-point response. The revised manuscript is substantially improved, and most of my prior concerns have been addressed satisfactorily. In particular, the authors have clarified the time-varying exposure framework, reported the number and timing of participants who converted from SARS-CoV-2 negative to positive during follow-up, added incidence rates, expanded the description of missing data and imputation, included an additional model adjusted for BMI and education, accounted for site-level differences using a frailty term, and appropriately tempered the interpretation of the null findings.

I have no remaining major concerns regarding the scientific soundness of the work. However, I recommend that a few minor issues be corrected before acceptance:

1. The conclusion appears to retain an outdated statement that incident diabetes occurred in 3.7% of participants over an average of roughly 1,000 days of follow-up. This is inconsistent with the revised Results and Table 1, which report incident diabetes in 6.5% of SARS-CoV-2 positive and 6.7% of SARS-CoV-2 negative participants, with median follow-up of approximately 4 years and corresponding incidence rates of 16.1 and 21.2 cases per 1,000 person-years. Please harmonize the conclusion with the revised results.

2. The description of Figure 2 and the Kaplan-Meier analysis remains somewhat ambiguous. The response states that the Kaplan-Meier curves are descriptive and stratified by baseline SARS-CoV-2 status, whereas the manuscript refers to “extended Kaplan-Meier” curves and also emphasizes time-varying SARS-CoV-2 exposure. Please clarify whether Figure 2 represents baseline-stratified descriptive curves or a time-varying survival display, and ensure that the figure caption, Methods, and Results are aligned.

3. Please correct remaining minor typographical and formatting errors. For example, Table 1 reports “103 (125%)” for one education category, which appears to be a missing decimal point. The conclusion also contains minor grammatical issues, such as “current clinical practice, which advise.”

After these minor corrections, I believe the manuscript will be suitable for publication.

Kind regards.

**********

7. PLOS authors have the option to publish the peer review history of their article (what does this mean?). If published, this will include your full peer review and any attached files.

If you choose “no”, your identity will remain anonymous but your review may still be made public.

**Do you want your identity to be public for this peer review?**  For information about this choice, including consent withdrawal, please see our Privacy Policy.

Reviewer #2: No

**********

[NOTE: If reviewer comments were submitted as an attachment file, they will be attached to this email and accessible via the submission site. Please log into your account, locate the manuscript record, and check for the action link "View Attachments". If this link does not appear, there are no attachment files.]

To ensure your figures meet our technical requirements, please review our figure guidelines: https://journals.plos.org/plosone/s/figures

You may also use PLOS’s free figure tool, NAAS, to help you prepare publication quality figures: https://journals.plos.org/plosone/s/figures#loc-tools-for-figure-preparation.

NAAS will assess whether your figures meet our technical requirements by comparing each figure against our figure specifications.

# Author comment: Author response to Decision Letter 2

**DOI:** 10.1371/journal.pone.0351992.r004

28 May 2026

28 May 2026

Dear editors:

Thank you for your thoughtful review and for the opportunity to revise this manuscript. We respond to the reviewers’ comments point-by-point below.

Great gratitude,

Luke

Pandora Lucrezia “Luke” Wander, MD, MS, FACP

Associate Professor of Medicine

Adjunct Associate Professor of Epidemiology & Public Health Genetics

University of Washington

Staff Physician

Veterans Affairs Puget Sound Health Care System

Attachment Submitted filename: response to reviewers_062826.docx

# Peer review: Decision Letter 2

**DOI:** 10.1371/journal.pone.0351992.r005

**Authors:** Leonardo Roever, Felix Bongomin, Felix Bongomin

3 Jun 2026

Association of SARS-CoV-2 infection with incident diabetes among U.S. Veterans in a prospective longitudinal cohort

PONE-D-25-67341R2

Dear Dr. Wander,

We’re pleased to inform you that your manuscript has been judged scientifically suitable for publication and will be formally accepted for publication once it meets all outstanding technical requirements.

Within one week, you’ll receive an e-mail detailing the required amendments. When these have been addressed, you’ll receive a formal acceptance letter and your manuscript will be scheduled for publication.

An invoice will be generated when your article is formally accepted. Please note, if your institution has a publishing partnership with PLOS and your article meets the relevant criteria, all or part of your publication costs will be covered. Please make sure your user information is up-to-date by logging into Editorial Manager at Editorial Manager® and clicking the ‘Update My Information' link at the top of the page. For questions related to billing, please contact billing support.

If your institution or institutions have a press office, please notify them about your upcoming paper to help maximize its impact. If they’ll be preparing press materials, please inform our press team as soon as possible -- no later than 48 hours after receiving the formal acceptance. Your manuscript will remain under strict press embargo until 2 pm Eastern Time on the date of publication. For more information, please contact onepress@plos.org.

Kind regards,

Felix Bongomin, MB ChB, Ph.D., FECMM

Academic Editor

PLOS One

Additional Editor Comments (optional):

Reviewers' comments:

Reviewer's Responses to Questions

**Comments to the Author**

1. If the authors have adequately addressed your comments raised in a previous round of review and you feel that this manuscript is now acceptable for publication, you may indicate that here to bypass the “Comments to the Author” section, enter your conflict of interest statement in the “Confidential to Editor” section, and submit your "Accept" recommendation.

Reviewer #2: All comments have been addressed

**********

2. Is the manuscript technically sound, and do the data support the conclusions?

The manuscript must describe a technically sound piece of scientific research with data that supports the conclusions. Experiments must have been conducted rigorously, with appropriate controls, replication, and sample sizes. The conclusions must be drawn appropriately based on the data presented.

Reviewer #2: Yes

**********

3. Has the statistical analysis been performed appropriately and rigorously?

Reviewer #2: Yes

**********

4. Have the authors made all data underlying the findings in their manuscript fully available?

The PLOS Data policy requires authors to make all data underlying the findings described in their manuscript fully available without restriction, with rare exception (please refer to the Data Availability Statement in the manuscript PDF file). The data should be provided as part of the manuscript or its supporting information, or deposited to a public repository. For example, in addition to summary statistics, the data points behind means, medians and variance measures should be available. If there are restrictions on publicly sharing data—e.g. participant privacy or use of data from a third party—those must be specified.

Reviewer #2: No

**********

5. Is the manuscript presented in an intelligible fashion and written in standard English?

PLOS ONE does not copyedit accepted manuscripts, so the language in submitted articles must be clear, correct, and unambiguous. Any typographical or grammatical errors should be corrected at revision, so please note any specific errors here.

Reviewer #2: Yes

**********

6. Review Comments to the Author

Please use the space provided to explain your answers to the questions above. You may also include additional comments for the author, including concerns about dual publication, research ethics, or publication ethics. (Please upload your review as an attachment if it exceeds 20,000 characters)

Reviewer #2: (No Response)

**********

7. PLOS authors have the option to publish the peer review history of their article (what does this mean?). If published, this will include your full peer review and any attached files.

If you choose “no”, your identity will remain anonymous but your review may still be made public.

**Do you want your identity to be public for this peer review?**  For information about this choice, including consent withdrawal, please see our Privacy Policy.

Reviewer #2: No

**********

# Editor report: Acceptance letter

**DOI:** 10.1371/journal.pone.0351992.r006

**Authors:** Leonardo Roever, Felix Bongomin, Felix Bongomin

PONE-D-25-67341R2

PLOS One

Dear Dr. Wander,

I'm pleased to inform you that your manuscript has been deemed suitable for publication in PLOS One. Congratulations! Your manuscript is now being handed over to our production team.

At this stage, our production department will prepare your paper for publication. This includes ensuring the following:

* All references, tables, and figures are properly cited

* All relevant supporting information is included in the manuscript submission,

* There are no issues that prevent the paper from being properly typeset

You will receive further instructions from the production team, including instructions on how to review your proof when it is ready. Please keep in mind that we are working through a large volume of accepted articles, so please give us a few days to review your paper and let you know the next and final steps.

Lastly, if your institution or institutions have a press office, please let them know about your upcoming paper now to help maximize its impact. If they'll be preparing press materials, please inform our press team within the next 48 hours. Your manuscript will remain under strict press embargo until 2 pm Eastern Time on the date of publication. For more information, please contact onepress@plos.org.

You will receive an invoice from PLOS for your publication fee after your manuscript has reached the completed accept phase. If you receive an email requesting payment before acceptance or for any other service, this may be a phishing scheme. Learn how to identify phishing emails and protect your accounts at https://explore.plos.org/phishing.

If we can help with anything else, please email us at customercare@plos.org.

Thank you for submitting your work to PLOS ONE and supporting open access.

Kind regards,

PLOS ONE Editorial Office Staff

on behalf of

Dr. Felix Bongomin

Academic Editor

PLOS One
